# A Decade-Old Atlas of TMEM (Transmembrane) Protein Family in Lung Cancer: Lessons Learnt and Future Directions

**DOI:** 10.3390/ijms27021120

**Published:** 2026-01-22

**Authors:** Siwei Zhang, Guojie Cao, Xuelin Hu, Chen Chen, Peng Chen

**Affiliations:** Experimental Center of Biochemistry and Molecular Biology, Faculty of Basic Medical Science, Xi’an Medical University, Xi’an 710021, China; zsw@xiyi.edu.cn (S.Z.);

**Keywords:** transmembrane (TMEM) protein, lung cancer, signaling pathways, Gene Expression Profiling Interactive Analysis 2 (GEPIA2)

## Abstract

A growing body of work has linked the dysregulation of transmembrane (TMEM) proteins to the proliferation, metastasis, drug resistance, and tumor microenvironment remodeling of lung cancer, the leading global cause of cancer mortality. Renamed members such as STING1 (stimulator of interferon response cGAMP interactor 1, TMEM173), ANO1 (anoctamin-1, TMEM16A), ORAI1 (ORAI calcium release-activated calcium modulator 1, TMEM142A), ORAI3 (TMEM142C), and NDC1 (NDC1 transmembrane nucleoporin, TMEM48) are among the most extensively studied ones. Mechanisms of TMEM dysregulation in lung cancer span the modulation of Ca^2+^ influx, lysosomal exocytosis, ferroptosis, Wnt and β-catenin signaling, and immune cell infiltration and immune checkpoint rewiring, among others. Epigenetic silencing and targetable fusions (i.e., TMEM106B-ROS1 and TMEM87A-RASGRF1) create DNA-level vulnerabilities, while miRNA sponges offer RNA-level druggability. A subset of studies revealed context-specific expression (endothelial, B cell, and hypoxic EV) that can be exploited to remodel the tumor microenvironment. One study specifically focused on how isoform-specific expression and localization of TMEM88 determine its functional impact on tumor progression. Yet for most TMEMs, only pre-clinical or early-phase data exist, with many supported by a single study lacking independent validation. This review brings together scattered evidence on TMEM proteins in lung cancer, with the aim of guiding future work on their possible use as biomarkers or therapeutic targets.

## 1. Introduction

Lung cancer remains the deadliest malignancy worldwide, as 2,480,301 individuals were identified with lung cancer, while the disease claimed the lives of 1,817,172 people in 2022 [1]. In China, lung cancer accounted for 1,060,600 new cases and 733,300 deaths in 2022, making it the leading cause of cancer incidence and mortality in both men and women [2].

Despite revolutionary advances in targeted therapies (e.g., epidermal growth factor receptor (EGFR), anaplastic lymphoma kinase (ALK), and ROS1 inhibitors) [3,4] and immune checkpoint blockade (ICB) [5,6], intrinsic and acquired resistance to therapies, metastatic dissemination, and tumor heterogeneity remain significant challenges [7,8,9,10,11]. Consequently, the identification of novel molecular drivers and therapeutic vulnerabilities remains an urgent priority.

Transmembrane (TMEM) proteins—defined by the presence of at least one hydrophobic segment that fully or partially spans a lipid bilayer [12]—are integral components of plasma and organelle membranes (mitochondria, endoplasmic reticulum (ER), Golgi, and lysosomes) [13,14]. Initially numbered rather than named on the basis of the predicted transmembrane domains in their sequences [15], most TMEM entries remain structurally and functionally poorly characterized because of experimental difficulties in extraction and purification [15,16]. As knowledge accrues, many are reannotated into specific families such as G protein-coupled receptors (GPCRs) or ion channels [12], but growing evidence already links their aberrant expression to tumorigenesis, metastasis, and immune dysregulation [17].

Over the past decade, high-throughput genomics, transcriptomics, proteomics, and single-cell technologies have accelerated the discovery of TMEM protein involvement in lung cancer. Yet, no comprehensive, decade-spanning review has systematically mapped the entire TMEM landscape in lung cancer. This review aims to summarize the current understanding of TMEM proteins in lung cancer, with a particular focus on those that have not yet been assigned alternative nomenclatures. In the process, we hope to provide a comprehensive overview of the less-explored members of this family, highlighting their putative therapeutic relevance and areas for future research.

## 2. From Characterized TMEM Proteins to Survival-Guided Shortlisting in Lung Cancer

Approximately 300 proteins are classified as members of the TMEM family according to the Human Protein Atlas. As the understanding of their functions and structures deepens, several TMEM proteins have been given new names and assigned to more precise categories, highlighting the evolving nature of TMEM protein classification.

### 2.1. STING1 or TMEM173

Stimulator of interferon response cGAMP interactor 1 (STING1), also known as TMEM173, is a well-known ER-resident adaptor that, after DNA sensing, traffics from ER to the Golgi or ER-Golgi intermediate compartment and can be rerouted to mitochondria, endosomes, lysosomes, or nuclei [18,19]. Here, it triggers type-I IFN (IFN-I) and cytokine production, yet it also modulates autophagy and cell death (e.g., apoptosis, ferroptosis, necroptosis, pyroptosis, mitotic, and immunogenic death) [20,21]. Given that IFN-I is pivotal for launching antitumor immunity, pharmacological STING agonists have entered early-phase clinical studies intended to ignite STING signaling within malignant cells or intratumoral dendritic cells, either as monotherapy or alongside conventional regimens [18].

### 2.2. ANO1 or TMEM16A

ANO1 (anoctamin-1), also known as TMEM16A, along with TMEM16B, TMEM16C, TMEM16D, TMEM16E, TMEM16F, TMEM16G, TMEM16H, TMEM16J, and TMEM16K, belongs to the anoctamin family. TMEM16A and B function as Ca^2+^-activated Cl^−^ channels [22]. TMEM16A governs smooth muscle contraction and pericyte tone, regulates epithelial secretion, and has been implicated in hypertension, stroke, overactive bladder, cystic fibrosis, COPD, Sjögren’s syndrome, and dry eye syndrome [23,24]. In tumors, although TMEM16A is frequently overexpressed, its effects are cell type-specific and mediated by distinct signaling pathways [24]. Overexpression of ANO1 is associated with tumor growth, metastasis, and poor prognosis in malignancies including lung cancer [25,26,27,28,29,30]. Importantly, ANO1 is druggable through small-molecule blockers [31,32] or non-coding RNAs [33,34], offering fresh therapeutic avenues.

### 2.3. ORAI Proteins

Three ORAI Ca^2+^ release-activated Ca^2+^ modulators—ORAI1 (TMEM142A), ORAI2 (TMEM142B), and ORAI3 (TMEM142C)—were initially classified in the TMEM family. These homologous mammalian channels are plasma-membrane Ca^2+^ channels whose concerted or distinct actions control Ca^2+^ homeostasis [35]. ORAI1 constitutes the archetypal store-operated calcium release-activated calcium (CRAC) channel that replenishes sarcoplasmic and ER calcium stores after the detection of Ca^2+^ depletion via stromal interaction molecules 1 (STIM1) and STIM2 proteins, forming the store-operated Ca^2+^ entry (SOCE) signaling cascade [35,36,37]. While ORAI1 operates ubiquitously, ORAI2 and ORAI3 confer SOCE only in selected tissues and can additionally form arachidonic acid-regulated Ca^2+^ (ARC) channels [35]. Through these mechanisms, the ORAI proteins couple the Ca^2+^ influx to proliferation, immune modulation, migration, and survival, which are pathways that become oncogenic when dysregulated [37,38,39]. In non-small cell lung cancer (NSCLC), ORAI1 is markedly overexpressed, accelerating tumor progression by amplifying PI3K–AKT–ERK (phosphoinositide 3-kinase, protein kinase B, and extracellular signal-regulated kinase) signaling [40], increasing PD-L1 (programmed death-ligand 1) -bearing exosome release, and blunting antitumor T-cell response [39]. ORAI3, a mammalian-specific homolog, can hetero-oligomerize with ORAI1 to form ARC channels or leukotriene-C4-gated Ca^2+^ channels [35]. Importantly, ORAI3 is preferentially upregulated in malignant cells of breast, prostate, lung, and gastrointestinal origin but remains low in non-tumor tissue [35]. In several neoplasms, ORAI3 appears to supersede ORAI1 in functional relevance. Estrogen receptor-positive breast cancer and NSCLC cells rely predominantly on ORAI3 for SOCE, with ORAI1 playing a secondary role [35,41]. In prostate cancer, ORAI3 overexpression drives proliferation via ORAI1 and ORAI3 heteromers [41]. Lung adenocarcinoma (LUAD) exhibits pronounced ORAI3 upregulation relative to matched non-tumor tissue [42]. High-grade tumors show even greater ORAI3 abundance, which intensifies SOCE, accelerates G1–S transition through cyclin D1/CDK4 and Akt phosphorylation [42,43,44], and correlates with metastasis and shortened overall and metastasis-free survival in NSCLC tumors [42,43]. Moreover, ORAI3 enrichment sustains cancer stem cell traits and platinum resistance in NSCLC, whereas its pharmacological or genetic silencing restores chemosensitivity and reduces stemness markers [43]. Orai3 expression and function are under microRNA control; microRNA (miR)-18a and -18b act as positive regulators, whereas miR-34a represses the channel [45]. When ORAI1 is forcibly overexpressed, it paradoxically suppresses EGF-driven proliferation by dampening SOCE, blocking ERK and Akt signaling, and inducing G0 and G1 arrest [38]. Collectively, elevated expression of both ORAI1 and ORAI3 underpins cancer hallmarks across diverse tumor types, positioning them as prognostic indicators and actionable therapeutic targets in malignancies, including lung cancer.

### 2.4. NDC1 or TMEM48

Nuclear division cycle 1 homolog (NDC1 or TMEM48), a transmembrane nucleoporin that resides at the nuclear pore complex (NPC) and the inner nuclear envelope [46,47,48], is markedly upregulated in hepatocellular carcinoma [48,49], NSCLC [46,50], cervical cancer [51], and pancreatic cancers [52]. In contrast, in colorectal cancer, high NDC1 expression is associated with reduced tumor aggressiveness and favorable prognosis [53], highlighting a context-dependent role. Functionally, NDC1 facilitates NPC assembly to promote nuclear envelope formation and nuclear growth [47], and it modulates immune cell infiltration in tumor microenvironment [48,52,53]. NDC1 expression is therefore an independent predictor of prognosis [46,48,49,52]. In NSCLC, NDC1 suppresses NSCLC cell proliferation, adhesion, migration, and invasion in vitro while inhibiting tumorigenesis in vivo [46]. Additionally, NDC1 depletion induces G1-phase cell cycle arrest and promotes apoptosis in NSCLC [46]. Gene set enrichment analysis suggests that NDC1 influences the cell cycle pathway, supported by the downregulation of key genes such as cyclin B1, CDK1, CDC6, PCNA, and RCF4 following its knockdown [46]. Gene-silencing strategies—miR-421 [50] or siRNA-mediated knockdown [46,49]—can induce apoptosis and increase chemo-sensitivity, establishing NDC1 as a promising, though still pre-clinical, therapeutic target across multiple solid tumors, including lung cancer.

### 2.5. Other Renamed TMEMs in Lung Cancer

Research on the functions of other TMEM proteins has also made significant progress. TMEM2L, now known as cell migration-inducing and hyaluronan-binding protein (CEMIP), has been associated with cancer metastasis [54,55]. Vacuole membrane protein 1 (VMP1 or TMEM49) [56,57] and DNA damage regulated autophagy modulator 2 (DRAM2 or TMEM77) are essential autophagy-related proteins [58,59,60]. ER membrane protein complex subunit 6 (EMC6 or TMEM93) [61,62] and eva-1 homolog A (EVA1A or TMEM166) [63,64] have also been implicated in autophagy regulation. Notably, DRAM2 and EMC6 fuel lung cancer progression [65,66], whereas EVA1A suppresses tumor growth by simultaneously triggering autophagy, apoptosis, and G2 and M cell cycle arrest [64]. Additionally, plasmanylethanolamine desaturase-1 (PEDS1 or TMEM189) promotes tumorigenesis in gastric and breast cancers by inhibiting autophagosome formation [67] and autophagy-dependent ferroptosis [67,68,69,70]. PEDS1 is frequently upregulated in multiple malignancies, including LUAD, and serves as an independent risk factor for poor prognosis, partly by fostering an immunosuppressive tumor microenvironment [71]. IGF-like family receptor 1 (IGFLR1 or TMEM149) seems to relate to tumor-immune cell infiltration [72,73]. TMEM118 (ring finger protein, transmembrane 2 (RNFT2)) was identified as an oncogene [74,75,76]. TMEM113, namely WD repeat domain 82 (WDR82), is now known as an essential component or adaptor for transcription-termination pathways [77,78,79].

The canopy family currently comprises four proteins—CNPY1, CNPY2, CNPY3, and CNPY4—that share a conserved structural scaffold and were first identified as modulators of fibroblast growth factor (FGF) signaling [80]. Among them, only CNPY2 was initially annotated as TMEM4 [80,81] and characterized as an angiogenic factor driving neovascularization [80,82]. CNPY2 is overexpressed in multiple tumor types, where its abundance correlates with poor prognosis by promoting proliferation, invasion, and tumor microenvironment remodeling [81,82]. Similarly, within the diverse animal opsins—GPCRs that bind retinal to form photopigments—Opn5 is the sole member that was formerly designated TMEM13 [83].

The renamed TMEMs are listed in Appendix A Table A1, which provides their full names and aliases, as well as the log-rank *p* values for both progression-free survival (PFS) and overall survival (OS), calculated in Gene Expression Profiling Interactive Analysis 2 (GEPIA2) using the default FPKM dataset for LUAD and lung squamous cell carcinoma (LUSC) (i.e., without the optional “normalize by gene length” setting). For each gene, the result obtained with the cut-off—median (M) or quartile (Q)—that yielded the lower *p* value is reported. The cut-off used is indicated in the table.

### 2.6. TMEM Survival Association

Using the GEPIA2 database, we screened for TMEM genes whose expression significantly correlated with PFS or OS. For each TMEM, survival association was assessed independently for PFS and OS across four datasets—the default dataset (without the optional “normalized by gene” setting), GAPDH-normalized, ACTB-normalized, and GNAS-normalized—yielding eight *p* values per gene.

A TMEM is eligible for Table 1 only if, for at least one endpoint (PFS or OS), the same directional association (high expression linked to longer survival) is significant (*p* < 0.05) in ≥2 of the 4 analyses and no contradictory trend (high expression linked to shorter survival) was observed in any of the eight tests.

Conversely, Table 2 lists the genes meeting the mirror criterion: low expression consistently associated with longer survival in ≥2 of the 4 analyses for PFS or OS, again without any conflicting result in the remaining tests.

Any TMEM whose high- and low-expression strata gave opposing significant results for the same endpoint or whose significance direction differed among the four datasets was excluded from both tables.

To maintain consistency with the main text criteria, we provide the content of Table A2 as a non-filtered reference catalogue. In this table, a TMEM is listed if either (1) the survival analysis for LUAD and LUSC (using the default dataset) attained statistical significance (*p* < 0.05) for PFS or OS or (2) the same gene reached significance in at least two of the three normalized comparisons (GAPDH, ACTB, or GNAS) for the same endpoint, irrespective of whether the direction of association was concordant across datasets. Consequently, genes that showed contradictory trends (e.g., high expression associated with prolonged survival in one normalization but with shortened survival in another) are retained in Appendix A Table A2 but are deliberately excluded from the rigorously curated Table 1 and Table 2 in the main text.

Gene Expression Omnibus (GEO), The Cancer Genome Atlas (TCGA), and the European Genome-phenome Archive (EGA) have been utilized across multiple studies, suggesting that TMEMs such as TMEM213 [84], TMEM245 [85], and TMEM229A [86] may be associated with lung cancer prognosis. The findings from some of these studies were further validated through in vitro and in vivo experiments, as well as analyses of clinical tumor and tissue samples. The TMEMs supported by such evidence are discussed below.

## 3. TMEM Proteins with Tumor-Suppressive Roles in Lung Cancer

In the context of lung cancer, several TMEM proteins have been identified as potential tumor suppressors, exhibiting significant downregulation in tumor tissues compared with normal counterparts. These proteins, through their distinct mechanisms, contribute to the inhibition of tumor metastasis, proliferation, and invasion, highlighting their potential as diagnostic and prognostic biomarkers as well as putative therapeutic targets in lung cancer (Table 3).

### 3.1. TMEM8B

The TMEM8B-a protein represents the predominant and lengthier isoform encoded by the TMEM8B gene. It has been identified as a suppressor of tumor metastasis in cancers such as nasopharyngeal carcinoma and lung cancer [87]. Research indicates that in numerous cell lines derived from these cancers, the TMEM8B-a protein undergoes rapid degradation through the proteasome pathway [87]. This process is facilitated by ezrin; however, interestingly, degradation occurs without the protein first being ubiquitinated [87].

### 3.2. TMEM17

Studies have shown that the expression levels of TMEM17 are markedly reduced in lung cancer tissues compared with adjacent normal tissue [88]. This lack of TMEM17 expression was significantly correlated with several negative clinical indicators, including poorer tumor differentiation, more advanced disease stages, the presence of lymph node metastasis, and an overall worse patient prognosis [88]. Mechanistic investigations revealed that artificially increasing TMEM17 expression led to a decrease in phosphorylated ERK and its downstream targets, p-P90RSK and Snail [88]. Concurrently, it increased the levels of proteins associated with cell adhesion, such as occludin and ZO-1 (Zonula occludens-1), ultimately resulting in suppressed invasion and migration capabilities of the cancer cells [88].

### 3.3. TMEM52B

TMEM52B is also metastasis-related. Higher TMEM52B expression is linked to better prognosis and coincides with preserved E-cadherin across several solid tumors, including lung tumors [105]. A related molecular mechanism was found in colon cancer cells [105]. The knockdown of TMEM52B promotes epithelial-mesenchymal transition (EMT), invasion, and cell survival in vitro, and it enhances tumor growth and circulating tumor cell levels in vivo. These effects are mediated through the destabilization of E-cadherin, which liberates β-catenin and generates soluble E-cadherin fragments that activate EGFR and its downstream ERK1 and 2, JNK (c-Jun N-terminal kinase), and AKT signaling pathways [105].

The molecular mechanisms underlying TMEM52B loss in lung cancer remain largely unexplored, whereas TMEM100 has been extensively investigated in pulmonary tumors. TMEM100 is consistently downregulated in NSCLC tissues and associated with poorer patient prognosis [89,91,92,93]. TMEM100 is differentially expressed in tumors compared with the adjacent normal tissues [106]. TMEM100 and four other genes (DCUN1D5, PSMC4, TFAM, and THRA) were consistently identified as prognostic markers through alternative polyadenylation events in LUAD across distinct ethnic populations (i.e., East Asia and USA) [107].

### 3.4. TMEM139

TMEM139 binds directly to E-cadherin both on the plasma membrane and within focal adhesions [96]. By blocking the lysosomal degradation pathway of E-cadherin, TMEM139 suppresses EMT, the migration and invasion capabilities of NSCLC cells in both cellular models, as well as the metastasis of NSCLC cells in animal models [96].

### 3.5. TMEM100

The TMEM100 expression level was discovered to be decreased in both NSCLC tissues (TCGA database, GEO database, and Oncomine database) [91,92,93] and cell lines compared with normal lung tissues and the immortalized human bronchial epithelial cell line, and it was observed to be negatively associated with the tumor, node, and metastases (TNM) stage (TCGA database) and positively associated with prognosis (Kaplan–Meier Plotter database) [91,92,93]. TMEM100 inhibited tumor cell proliferation and promoted cell apoptosis and autophagy in NSCLC cells [91,92]. Inhibiting autophagy using bafilomycin A1 significantly enhanced TMEM100-induced apoptosis to compensate for cell death [91].

The downregulation of TMEM100, a downstream effector of ALK1 signaling that is normally restricted to the vasculature of developing organs but selectively maintained in adult lungs [108], is already significant at the earliest stages of LUAD [90]. Specifically, Kadonaga et al. demonstrated that its mRNA is significantly lower in noninvasive lesions than in adjacent histologically normal lungs (aNLs), and this repression persists as LUAD advances [90]. Immunohistochemistry (IHC) in an independent validation set confirmed substantially reduced TMEM100 protein levels in both noninvasive lesion and invasive lesion samples relative to an aNL, while a modest, non-significant rise in the invasive lesion samples was observed, underscoring the persistent suppression thought to drive the initiation and early evolution of LUAD independent of the EGFR mutation status [90]. TCGA data analysis further showed that early-stage LUAD patients with preserved high TMEM100 expression experienced significantly longer relapse-free survival than the patients with low TMEM100 expression (*p* = 0.025), although OS was unaffected. These observations reinforce the earlier finding by Frullanti et al. that TMEM100 mRNA expression is downregulated in LUAD compared with distant non-involved lung tissue [109] and implicate TMEM100 loss as both a tumor-initiating event and a prognostic indicator of recurrence risk.

TMEM100 is a potent metastasis suppressor as it inhibits the Wnt/β-catenin signaling pathway, thereby reversing EMT—as evidenced by increased epithelial markers and decreased mesenchymal markers—which consequently reduces cell migration, invasion, and proliferation. In vivo, its overexpression suppresses metastatic burden and prolongs survival [93]. When tracing its upstream regulator, HDAC6 transcriptionally represses TMEM100, thereby reactivating Wnt and β-catenin signaling and EMT to promote metastasis [93].

Wang et al. [93] and Hong et al. [94] both emphasized the role of TMEM100 in NSCLC metastasis, yet they offered differing interpretations regarding the molecular mechanisms by which TMEM contributes to tumor metastasis [93,94]. Hong et al. [94] indicated that in LUAD tissues, miR-421 was found to be markedly overexpressed, whereas both circ_0000567 and TMEM100 were significantly downregulated compared with adjacent normal tissues [94]. Functionally, circ_0000567 was shown to elevate TMEM100 expression and suppress cell migration and invasion [94]. Conversely, miR-421 promoted these malignant behaviors [94]. Mechanistically, circ_0000567 acts as a molecular sponge for miR-421, thereby preventing miR-421 from binding to and degrading TMEM100 mRNA in LUAD cells [94].

### 3.6. TMEM106A

Low expression of TMEM106A was detected in human NSCLC tissues and NSCLC cell lines compared with adjacent normal lung tissues and the human bronchial epithelial cell line (HBE1), respectively [95]. TMEM106A overexpression suppresses proliferation, migration, invasion, and EMT while promoting apoptosis in NSCLC cells. These effects are mediated through inhibition of the PI3K/Akt/NF-κB signaling pathway. The use of SC79, an activator of PI3K and Akt signaling, partially reversed the TMEM106A-induced suppression of EMT in A549 cells, confirming the involvement of the PI3K/Akt/NF-κB signaling pathway in TMEM106A-suppressed EMT [95].

### 3.7. TMEM164

Compared with non-tumor lung tissues and the normal human bronchial epithelial cell line BEAS-2B, TMEM164 expression is markedly reduced in LUAD tumors and in the LUAD cell lines A549 and NCI-H358, as verified in both the Human Protein Atlas (HPA) database and clinical specimens [97]. Low expression of TMEM164 exhibits poor prognosis in LUAD cases [97]. TMEM164 overexpression inhibits the proliferation, invasion, and metastasis of LUAD cells both in vitro and in vivo [97].

TMEM164 acts as an upstream regulator that promotes ferroptosis in LUAD cells by enhancing ATG5-dependent autophagosome formation [97]. The presence of TMEM164 strengthens the ATG5–ATG16L1 interaction under ferroptotic stress, driving autophagy that preferentially degrades glutathione peroxidase 4 (GPX4) over other substrates [97]. Consequently, TMEM164-mediated autophagy ultimately induces ferroptosis mainly through GPX4 degradation, highlighting a selective pathway distinct from canonical autophagy components [97]. Independent work by Qiao and Chen [98] corroborated the ferroptosis-promoting role of TMEM164; TMEM164 knockdown in A549 cells lowered lipid peroxidation, preserved GSH levels, reduced free Fe^2+^ and MDA accumulation, and increased cell viability, collectively indicating attenuated ferroptotic death. They further identified TRIM59 as a negative post-translational regulator of TMEM164; TRIM59 physically ubiquitinates TMEM164 and targets it for proteasomal degradation [98]. Inhibition of TRIM59 stabilized TMEM164, restored its pro-autophagic activity, and resensitized LUAD cells to autophagy-dependent ferroptosis [98].

In addition, TMEM164 significantly influences the immune microenvironment in LUAD by promoting CD8+ T cell-mediated cytotoxicity, proliferation inhibition, and apoptosis of LUAD cells. Additionally, TMEM164 expression enhances the efficacy of anti-PD-1 (programmed cell death protein 1) therapy in mouse models, where combination treatment leads to suppressed tumor growth without affecting body weight. This sensitization is associated with increased ferroptosis, as indicated by elevated levels of ferroptosis-related proteins, lipid peroxidation markers, and oxidative stress products [97].

### 3.8. TMEM176A

TMEM176A and B (MS4B1 and 2) are the membrane-spanning 4A (MS4A) family proteins [110]. Although an earlier study reported elevated TMEM176A protein levels in lung tumors versus normal tissue [111], Li et al. [99] later showed that promoter hypermethylation downregulates TMEM176A in NSCLC, whereas its re-expression induces apoptosis and G2 and M arrest and suppresses migration, invasion, proliferation, colony formation, and H1299 xenograft growth [99]. Methylation-induced loss of TMEM176A activates ERK signaling and sensitizes cells to the Ataxia telangiectasia mutated (ATM) inhibitor AZD0156, indicating that TMEM176A is not part of the ATM or ATR (AT and Rad3-related) pathway [99].

### 3.9. TMEM229A

Like TMEM176A, TMEM229A promotes cell proliferation, colony formation, EMT, migration, and invasion, potentially through the ERK signaling pathway [86].

### 3.10. TMEM196

TMEM196 expression, predominantly localized in the cytoplasm, was significantly downregulated at both the mRNA and protein levels in lung cancer cell lines and human lung tumor tissues (including LUAC and LUSC) compared with normal HBE cells and adjacent normal tissues [100,101]. Consistent downregulation of TMEM196 was confirmed in lung cancer tissues in comparison with adjacent normal tissues in both the TCGA database and independent lung cancer patient cohorts analyzed via IHC [100,101]. Low TMEM196 expression was associated with worse prognosis than high TMEM196 expression, and TMEM196 served as an independent prognostic biomarker for lung cancer [100,101,102].

TMEM196 downregulation or inactivation is associated with promoter methylation in both rat and human primary lung cancer tissues and cell lines [101]. TMEM196 hypermethylation was observed in chemically induced rat lung lesions, human lung cancer tissues, and cell lines but not in normal lung tissues or HBE cells [101]. TMEM196 hypermethylation in lung cancer tissues was later verified in the TCGA database compared with adjacent normal tissues [102]. The frequency of TMEM196 methylation was positively correlated with the pathological severity of lung carcinogenesis [101]. Furthermore, subsequent studies by Liu et al. identified TMEM196 hypermethylation as a highly sensitive and noninvasive biomarker detectable in plasma and sputum, showing strong concordance with primary lung tumors and a significant association with patient age and cancer type [102]. The association between TMEM196 hypermethylation and poor prognosis was verified by the TCGA database, especially in cases with TNM I and II [102].

Gain and loss-of-function studies consistently demonstrate that TMEM196 suppresses tumor metastasis and progression both in vitro and in vivo [100,101]. Mechanistically, TMEM196 induces apoptosis by upregulating the pro-apoptotic factor Bax and the cell cycle inhibitor p21 while downregulating the proliferation drivers cyclin D1 and c-Myc and the promigratory adhesion molecules CD44 and β-catenin. Conversely, TMEM196 knockdown in HBE cells yields the opposite expression profile [101]. Chen et al. [100] confirmed the suppressive role of TMEM196 in lung cancer metastasis and revealed that this inhibition occurs through downregulation of β-catenin transcription within the Wnt/β-catenin pathway.

These studies pointed out the potential use of TMEM196 methylation and expression as novel early diagnostic and prognostic biomarkers for human lung cancers.

Growing evidence links TMEM family methylation to lung tumors. TMEM88 [104], TMEM176A [99] and TMEM196 [101] all display frequent hypermethylation in lung tumors, implying epigenetic silencing, whereas TMEM171 has been identified as 1 of 17 methylation driver genes whose integrative signature, combined with four CD8+ T cell-associated genes, yields a robust prognostic index for early-stage LUAD patients [112].

## 4. TMEM Proteins Associated with Tumor Promotion in Lung Cancer

The following sections provide a comprehensive overview of various TMEM proteins and their roles in lung cancer. These proteins have been identified through extensive research and analysis, highlighting their potential as diagnostic or prognostic biomarkers and candidate therapeutic targets that still await druggability validation. Their functions and mechanisms are elucidated through studies involving clinical samples, cell lines, and experimental models (Table 4).

### 4.1. TMEM14A

GEPIA2 shows TMEM14A overexpression in LUAD and links high levels to shortened OS [114]. Silencing TMEM14A dampens mitochondrial ATP production and inhibits NSCLC proliferation [114]. Evidence suggests that circ_0003645 functions as an oncogenic circular RNA in NSCLC by acting as a sponge for miR-1179, thereby upregulating the expression of TMEM14A, a direct target of miR-1179 [113]. Experimental results confirmed an inverse correlation between circ_0003645 and miR-1179 levels, while TMEM14A was elevated in NSCLC tissues [113]. Functional rescue experiments conducted in NSCLC cell lines confirmed that the oncogenic effects of circ_0003645 are mediated through the miR-1179/TMEM14A axis [113]. TMEM14A suppression via circ_0003645 downregulation inhibits NSCLC cell proliferation, migration, and invasion, which could be partially reversed by overexpressing TMEM14A or inhibiting miR-1179 [113]. Conversely, the oncogenic phenotype induced by circ_0003645 overexpression was attenuated by either miR-1179 mimics or TMEM14A silencing [113]. Separately, receptor tyrosine kinase AXL transcriptionally upregulates TMEM14A via putative STAT (signal transducer and activator of transcription) and NF-κB sites in its 5′ promoter region [114].

### 4.2. TMEM33

Computational findings (GEPIA) and clinical LUAD specimens show TMEM33 mRNA and protein upregulation versus normal lung tissues. High levels predict poor OS and are associated with more lymph nodal metastasis and an advanced TNM stage [115]. Expression is also elevated in LUAD cell lines relative to BEAS-2B [115], while shRNA-mediated knockdown of TMEM33 curbs proliferation, spheroid formation, and invasion and expression of mesenchymal markers (vimentin and N-cadherin), whereas lithium chloride-activated Wnt and β-catenin signaling rescues these malignant phenotypes, placing TMEM33 upstream of this pathway [115].

TMEM33 knockdown cells exhibit reduced tumor aggressiveness in vivo, as evidenced by the decreased tumor weight and volume in the subcutaneous injection model, and they decreased the number of metastatic nodules in the lungs in the tail vein injection model [115]. Knockdown TMEM33 lowers Ki-67, SOX2 (SRY-box transcription factor 2), β-catenin, c-Myc and cyclin D1 [115], while miR-214-3p directly targets and negatively regulates TMEM33 in LUAD [115].

### 4.3. TMEM45A

TMEM45A expression rises with the tumor stage in vivo and exceeds levels seen in 2D or 3D cultures [116]. IHC confirmed high expression in LUAD tissues (n = 17) versus pneumothorax controls (n = 3) [117].

### 4.4. TMEM45B

The TCGA database and qPCR validation show TMEM45B upregulation in lung cancer, correlating with shorter OS [118]. Its silencing impairs proliferation, migration, and invasion in vitro, arrests cells at G1 and S, increases apoptosis, and retards xenograft growth by regulating CDK2 (Cyclin-dependent kinase 2), CDC25A (cell division cycle 25A), PCNA (proliferating cell nuclear antigen), Bcl-2, MMP-9 (matrix metalloproteinase-9), Twist (Twist family bHLH transcription factor 1), Snail (Snail family transcriptional repressor 1), Bax, and cleaved caspase-3 [118].

### 4.5. TMEM74

TMEM74 ranks among the 15 autophagy-related genes most upregulated in female LUAD and is associated with poor prognosis [132]. Protein overexpression is evident in tumors and cell lines, correlates with shortened survival [119], and is thought to propel tumor progression by enhancing autophagy [132].

### 4.6. TMEM98

The siRNA silencing of TMEM98 curbed proliferation, invasion, and migration of A549 and H460 cells and downregulated MMP-2, MMP-9, RhoC (Ras homolog family member C), and MTA1 (metastasis associated 1) [122], indicating that TMEM98 fuels NSCLC aggressiveness. Meanwhile, miR-29c-5p directly targets TMEM98 3′-UTR [121]. This exosomal miRNA is enriched in early-stage LUAD plasma, implicating the miR-29c-5p/TMEM98 axis in LUAD pathogenesis [121].

### 4.7. TMEM106B

GEPIA data link high TMEM106B mRNA expression to poor OS in lung cancer patients [133]. Machine learning analysis ranks TMEM106B at the top for separating obstructive sleep apnea (OSA) and lung cancer from controls [134].

In vivo CRISPR screening of 217 genes in non-metastatic 393P cells singled out TMEM106B as the strongest driver of lung cancer metastasis; its overexpression raises metastatic burden without altering primary tumor growth, whereas knockdown suppresses migration, invasion, and TGF-β-induced invasive structures in 3D cultures both in human and murine lung cancer cell lines [123]. Consistently, subcutaneous implantation of TMEM106B-depleted cells yielded primary tumors of comparable size to their controls but significantly suppressed lung metastasis [123].

Mechanistically, the lysosomal transmembrane protein TMEM106B is sufficient but not a necessary condition for the nuclear translocation of the transcriptional regulator TFEB (master regulator of lysosome synthesis and function), thereby upregulating the CLEAR network genes, including those encoding multiple cathepsins and lysosomal components. This drives the formation of enlarged, cathepsin-enriched lysosomes and facilitates calcium-dependent lysosomal exocytosis. Likewise, active cathepsins (such as B, D, H, and K) are secreted into the extracellular matrix, promoting invasion and metastasis; broad-spectrum or cathepsin-specific inhibitors reverse the phenotype [123].

Further supporting the functional significance of secreted proteases, a conditioned medium from TMEM106B-overexpressing cells significantly enhanced the invasive behavior of noninvasive parental cells, an effect blocked by protease inhibition [123].

Clinically, TMEM106B amplification or overexpression in lung adenocarcinoma independently predicts shorter DFS and OS, positioning it as both prognostic marker and therapeutic target [123].

### 4.8. TMEM116

Both the GEPIA dataset and clinical samples show that TMEM116 expression is higher in LUAD and LUSC than in normal lung tissues [124]. In clinical samples and benzo(a)pyrene-induced lung cancer mouse models, TMEM116 is expressed in tumor areas lacking airway epithelial or mesenchymal cell characteristics (CC10/β-tubulin and α-SMA) [124]. In non-tumor areas, it is expressed in airway and alveolar epithelial cells. Western blot analysis confirmed its higher expression in A549 and H1299 cell lines compared with the 16HBE cell line [124]. High TMEM116 expression is associated with shorter OS in LUAD patients but not in LUSC patients. In A549 cells, TMEM116 knockdown downregulates PDPK1 (3-phosphoinositide-dependent protein kinase 1; historically referred to as PDK1) and suppresses proliferation, clonogenicity, invasion, and migration in vitro, as well as metastatic burden and subcutaneous tumor growth in vivo [124]. These effects are largely rescued by the PDPK1 activator PS48, indicating that PDPK1 is a major mediator of TMEM116-driven oncogenicity [124]. Downstream of PDPK1, the migration and invasion of A549 cells are partially mediated through the AKT/FOXO3A/TAp63 axis and its downstream targets, including polarity- and adhesion-related desmoplakin, par-3 family cell polarity regulator (PARD3), and E-cadherin [124].

### 4.9. TMEM158

Linear regression across cell line panels of SCLC and NSCLC identified TMEM158 as a candidate gene; its knockdown in cisplatin-resistant PC-9 cells restored drug sensitivity [125]. TMEM158 is consistently upregulated in LUAD lines (A549, PC9, and H1650) versus non-malignant BEAS-2B [126]. High TMEM158 levels drive migration, EMT [127], proliferation, cell cycle progression, and invasion while suppressing apoptosis, and knockdown reverses these phenotypes [126].

Mechanistically, TMEM158 interacts directly with TWIST1 to activate PI3K and AKT signaling [126]. Hypoxia induces TMEM158 in an HIF-1α-dependent manner [127]. TCGA, GSE140797, qRT-PCR, and western blot analysis uniformly showed TMEM158 overexpression in LUAD tumors versus healthy tissues [126,127]. An elevated transcript predicted shorter OS [126,127] and disease-specific survival (DSS) in LUAD patients [126], correlating with pack-years and nodal metastasis [126] and being highest in advanced-stage tumors [127].

### 4.10. TMEM160

TMEM160 is upregulated in LUAD versus matched non-tumor tissue and accumulates in both the nucleus and cytoplasm, shuttling with the mitotic cycle (nuclear in interphase, cytoplasmic from pro-metaphase to telophase, and chromatin-associated yet still cytoplasmic at cytokinesis). Its abundance is also higher in A549 than in BEAS-2B cells, and silencing the protein inhibits proliferation and migration in vitro while reducing the tumor volume in vivo. Co-IP-MS followed by Gene set enrichment analysis (GSEA) links the TMEM160 interactome to apical junctions, xenobiotic metabolism, glycolysis, EMT, ROS handling, UV-DNA damage response, p53 signaling, and the mitotic spindle, with strong enrichment in nucleocytoplasmic transport (NUP50, KPNA6, and SRRM1). Public database mining connects TMEM160 to DNA replication, amino acid biosynthesis, and cell cycle control [128].

### 4.11. TMEM176B

TMEM176B is frequently upregulated in LUAD compared with adjacent normal or LUSC tissues, and its abundance rises with the stage; high expression predicts shorter OS. Forced expression of TMEM176B in LUAD cell lines accelerates proliferation, invasion, migration, and cell–matrix adhesion in vitro, and it fosters subcutaneous tumor growth in vivo. Mechanistically, TMEM176B amplifies FGFR1 (fibroblast growth factor receptor 1) signaling to activate JNK and downstream vimentin and Snail, thereby driving EMT. These oncogenic effects are reversed by FGFR or JNK inhibitors, positioning TMEM176B as a stage-associated promoter of LUAD aggressiveness that operates through the FGFR1–JNK–vimentin/Snail axis [129].

### 4.12. TMEM179

qRT-PCR and western blot analysis revealed higher expression of TMEM179 in A549 cells compared with BEAS2B cells. Genetic comparisons between NSCLC and normal tissues in the TCGA database identified three significantly different lncRNAs (LINC00968, lnc-FAM92A-9, and lnc-PTGFR-1) and six mRNAs (CTCFL, KRT5, LY6D, TMEM, GBP6, and TMEM179), with potential therapeutic significance in BEAS2B and A549 cells [135]. However, further validation in other cell lines and clinical settings is needed.

### 4.13. TMEM243

An early study conducted by Dorman et al. found out that TMEM243 emerged as 1 of 15 genes whose expression levels inversely correlates with growth inhibitory concentrations of paclitaxel and gemcitabine across breast cancer cell lines [136]. In a panel of lung cancer cell lines with varying degrees of paclitaxel resistance, TMEM243 was also recognized as a paclitaxel resistance inducer whose expression rises in parallel with the loss of the tumor suppressor protein alpha-2-macroglobulin (A2M) [131]. Re-expression of A2M represses TMEM243 (along with ABCB1 and ID1), thereby resensitizing resistant cells to paclitaxel. Thus, TMEM243 is a key downstream effector driving paclitaxel resistance in lung cancer and is negatively regulated by A2M [131].

## 5. Dual Roles of TMEM88 in Lung Cancer

### 5.1. Tumor Suppressor

According to the study by Jang et al., low expression of miR-708 is associated with better OS, and miR-708 is expressed at higher levels in LUAD tissues compared with normal lung tissues [103]. Analysis of the TargetScan and MicroCosm databases further revealed that TMEM88 is significantly downregulated in tumors with high miR-708 expression relative to those with low expression [103]. This regulatory relationship was confirmed with a luciferase assay, which demonstrated that miR-708 inhibits TMEM88 transcription [103]. The overexpression of miR-708 was shown to reduce TMEM88 expression in a dose-dependent manner [103]. Consistently, TMEM88 mRNA expression was found to be significantly lower in LUAD tissues compared with matched normal adjacent tissues. Moreover, higher TMEM88 expression was associated with improved OS, further supporting its role as a tumor suppressor negatively regulated by miR-708 [103].

Beyond miRNA-mediated repression, TMEM88 expression is also governed by promoter methylation. Notably, the trend reported by Ma et al. [104]—whereby low TMEM88 abundance fosters tumor progression—aligns with the earlier observation made by Jang et al. [103]. TMEM88 is hypermethylated in NSCLC, which was verified in an expanded cohort that promoter CpG-island methylation was markedly higher in tumors than in matched non-malignant lung tissue [104]. Hypermethylation of TMEM88 was inversely correlated with TMEM88 mRNA abundance [104]. Hypermethylation was significantly associated with larger tumor sizes and shorter OS, whereas the transcript level per se showed no prognostic value [104]. qRT-PCR validation of 201 tumor and 66 adjacent specimens confirmed underexpression of TMEM88 in cancerous tissue [104]. Pharmacologic demethylation restored TMEM88 expression, restrained proliferation, suppressed migration and invasion, and induced G2 and M arrest [104]. Analysis of TCGA data revealed only weak positive correlations between TMEM88 mRNA and Wnt pathway genes (DVL1, FZD4 and 5, and ROR1) [104], suggesting that the tumor-suppressive function of TMEM88 is largely independent of global Wnt signaling modulation.

### 5.2. Dual Roles of CRA-a

At the level of isoform-specific protein expression—and, critically, the subsequent subcellular distribution—the correlation between TMEM88 and tumor progression or patient survival is dramatically reshaped.

Zhang et al. [120] demonstrated that the function of TMEM88 in NSCLC is determined by its isoform type and subcellular localization. TMEM88, a potential two-transmembrane protein, interacts with disheveled-1, which is mainly involved in Wnt signaling. Two isoforms have been identified: CRA-a (isoform 1), which contains a C-terminal Val-Trp-Val (VWV) motif essential for binding to disheveled and inhibiting the canonical Wnt/β-catenin pathway, and CRA-b (isoform 2), which lacks this motif and shows no disheveled interaction [120].

In NSCLC tissues, TMEM88 is frequently overexpressed in the cytoplasm, and its elevated expression correlates with advanced TNM stages, lymph node metastasis, and poor OS [120]. In contrast, membrane-localized expression is less common [120]. The CRA-a isoform is significantly upregulated in NSCLC as well as in other cancers such as breast, colon, hepatocellular, and gastric carcinomas, while CRA-b remains unchanged [120].

Functionally, the subcellular localization of CRA-a dictates its role; when cytosolic, it promotes metastatic behaviors—through disheveled-dependent activation of the P38/GSK3β (glycogen synthase kinase 3)/ATF2 (activating transcription factor 2) axis, Snail stabilization, and downregulation of occludin and ZO-1—without affecting proliferation [120]. Conversely, membrane-localized CRA-a suppresses Wnt signaling, inhibits expression of cyclin D1, MMP-7, and c-Myc, and reduces tumor growth and metastasis in vitro and in vivo [120].

## 6. Less Characterized TMEMs with Predicative Values

### 6.1. TMEM92

TMEM92 is one of 14 core genes whose high expression (HR = 1.36) marks poor prognosis in LUSC. When ≥4 of these genes are aberrantly expressed, patients are stratified into a high-risk group with significantly shorter survival, linking TMEM92 to aggressive tumor biology and glycolytic reprogramming [137].

### 6.2. TMEM161A

TMEM161A is overexpressed in NSCLC compared with adjacent lung tissue and presents cross-reactive epitopes from Epstein–Barr virus and *E. coli*. This cross-reactivity may explain the presence of virus-specific T cells in tumor infiltrates and could be a feature of multiple cancers [138].

### 6.3. TMEM163

TMEM163 acts as a tumor-suppressive gene in LUAD; higher expression correlates with reduced mortality risk and is part of a four-gene signature (HLF–CHRDL1–SELENBP1–TMEM163) that robustly predicts better OS across four independent LUAD cohorts [139].

Lin et al. (2023) showed that taxifolin downregulates the signature of mouse counterparts of IIT (ITGAL (integrin subunit alpha L)-ITGAX (integrin subunit alpha X)-TMEM119), whose expression (i.e., stromal expressions of ITGAL and ITGAX and tumor expression of TMEM119 in NSCLC) predicts both ICB (immune checkpoint blockade therapy) benefit and mortality risk in NSCLC [140].

### 6.4. TMEM184A

TMEM184A forms a tightly correlated sub-network with KRT7 and ANKRD30A, and its expression—together with the mutational profile that includes TMEM184A—contributes to the gene signature that predicts a durable response to anti-PD-1 and PD-L1 therapy [141].

### 6.5. TMEM125

Although Yu et al. reported that TMEM125 mRNA is significantly higher in LUSC than in LUAD and normal lung tissue [142], a subsequent large-scale, multi-cohort study profiled TMEMs in LUAD for the first time.

A three-gene signature (TMEM125, TMEM164, and TMEM273) built from 477 TCGA tumors and validated in 731 external samples robustly stratifies patients (risk score = (−0.32738) × EXPTMEM273 + 0.25554 × EXPTMEM164 + (−0.29264) × EXPTMEM125) [17]. The high score independently predicts shortened OS and relapse-free survival. Mechanistically, the TMEM-based signature mirrors tumor proliferation, fatty acid metabolism, immune signaling, and global histone modification programs [17]. Specifically, high-risk tumors display H3K4me3 (histone H3 lysine 4 trimethylation) hypotrimethylation, reduced chemokine secretion, impaired macrophage chemotaxis, and broad immunosuppression, changes mediated, at least in part, by the aberrant expression of TMEM125, TMEM164, and TMEM273 [17]. Thus, these TMEMs not only serve as prognostic biomarkers but also highlight epigenetic and immune escape pathways driving LUAD progression.

## 7. Discordance Between GEPIA Survival Curves and Published Functional Findings

The above literature-reported, still-unnamed TMEMs are listed in Table 5. Their prognostic value was then tested in the two GEPIA2 lung cancer datasets: LUAD and LUSC. Distinct survival patterns emerged between the histotypes and in the combined set.

Although previous functional studies have proposed some TMEMs as tumor suppressors, GEPIA2 survival analyses yielded no association or even a paradoxical low expression, better prognosis pattern, except for TMEM8B, TMEM17, and TMEM213 (Table 5). TMEM17 showed high expression linked to longer disease-free survival (DFS) in the two datasets (LUAD + LUSC), yet within LUAD alone, a low TMEM17 level was associated with better survival. TMEM213 and TMEM8B displayed the expected positive correlation (high expression, better survival) exclusively in LUAD, whereas no association was detectable in LUSC or in the two datasets. TMEM125 displayed histotype-specific concordance; high levels predicted favorable survival in LUAD, whereas low levels were advantageous in LUSC and the two datasets. TMEM243, by contrast, showed better survival among patients with high expression in both LUAD and the two datasets.

Among the TMEMs previously characterized as oncogenic by functional assays, a considerable fraction exhibited GEPIA2-based survival trends that aligned with their tumor-promoting reputation (Table 5). TMEM33, TMEM45A, TMEM45B, TMEM74, TMEM98, and TMEM106B all displayed a “low expression, longer survival” pattern in LUAD or in the two datasets. However, TMEM14A and TMEM158 only partially recapitulated this trend—their low expression was linked to better survival in the LUAD dataset—while TMEM14A followed the expected pattern in LUAD yet showed high expression associated with improved prognosis in LUSC or the two datasets. TMEM125 displayed histotype-specific concordance; high levels predicted favorable survival in LUAD, whereas low levels were advantageous in LUSC and the two datasets. TMEM243, by contrast, showed better survival among patients with high expression in both LUAD and the two datasets.

The GEPIA2 Survival analysis only partially echoed the bench work narrative. TMEM100 is one example; it presented a “neutral” face in silico, where no Kaplan–Meier split reached significance in either LUAD or LUSC, although multi-database mining (TCGA, GEO, and Oncomine) and IHC showed that TMEM100 is consistently downregulated in tumors versus normal lung tissue, correlating with higher TNM stages and shorter relapse-free survival periods in NSCLC, especially for LUAC. Functional assays revealed that restoring TMEM100 suppresses the Wnt/β-catenin pathway, reverses EMT, inhibits proliferation, migration, and invasion, and promotes apoptosis and autophagy, establishing it as a metastasis suppressor and early recurrence risk biomarker in NSCLC.

Notably, GEPIA2 survival correlations often conflict with published findings. This discrepancy runs both ways. Biologically, post-transcriptional control (e.g., miRs) can uncouple mRNA abundance from the protein level. Isoform ambiguity and normalization artifacts can mask or mimic prognostic signals, while bulk RNA-seq averages malignant and non-malignant cells. With a TCGA median tumor purity of ≈60%, the cancer-autonomous signal may be masked or even inverted [143,144] Stage specificity adds a further layer; survival curves record late-stage disease, whereas a gate keeper required during early transformation can become dispensable once compensatory mutations accumulate, rendering its transcript level irrelevant or inversely associated with the outcome.

Methodologically, most functional studies rely on a single lung cancer cell line, arbitrary transfection efficiencies, and short-term proliferation and migration assays that lack the statistical power, duration, or phenotypic breadth needed to predict patient survival. GEPIA2, in contrast, captures the net effect of a gene across all disease stages and treatments, while published gain- or loss-of-function assays usually mirror early, untreated tumor behavior.

Taken together, the bulk survival snapshot from GEPIA2 and the Petri dish phenotype each illuminate only one side of the same story. Rather than treating either as stand-alone proof, their convergence should serve as a filter; agreement between survival curves and functional data upgrades a gene to priority status for purity-controlled validation, whereas discordance demands independent confirmation—through IHC or qPCR in patient cohorts coupled with single-cell or immune-adjusted models—before any therapeutic claim is advanced.

## 8. Genetic Alterations

There is available evidence on how genomic alteration of several TMEM family members rewire oncogenic signaling and how these alterations may be exploited clinically.

### 8.1. TMEM106B–ROS1 Fusion

A female patient with stage IV LUAD and no history of smoking was found to harbor an in-frame TMEM106B–ROS1 fusion; the predicted 540-aa chimeric protein retained the N terminal of TMEM106B but substituted its C terminus with amino acids 1881–2341 of ROS1 [145]. Although the N-terminal region of TMEM106B is capable of homo- or heterodimerization with other TMEM106 family members, it remains unclear whether this dimerization affects the function of the TMEM106B-ROS1 fusion protein. Moreover, despite the fusion protein retaining the intact kinase domain of ROS1, the article does not explicitly state that the TMEM106B-ROS1 fusion variant leads to the activation of ROS1. Therefore, while TMEM106B-ROS1 may be a targetable receptor-tyrosine kinase rearrangement, its potential as a therapeutic target requires further investigation.

### 8.2. TMEM87A-RASGRF1 Fusion

RNA sequencing of an “exceptional responder” to sunitinib treatment, a person who never smoked with metastatic NSCLC that experienced a 33-month partial remission, revealed a novel TMEM87A–RASGRF1 fusion [146]. This fusion transcript joins the N-terminal transmembrane domain of TMEM87A to the Cdc25-homology guanine exchange factor domain of RASGRF1 (RAS guanyl releasing factor 1), resulting in the deletion of the autoinhibitory Pleckstrin homology 1 (PH1) motif [146]. Consequently, the fusion protein constitutively activates RAS (rat sarcoma viral oncogene homolog) by loading GTP, leading to sustained ERK phosphorylation and activation of the MAPK (mitogen-activated protein kinase) pathway [146].

CRISPR-Cas9 knock-in of the TMEM87A–RASGRF1 fusion in NIH/3T3 fibroblasts induced anchorage-independent growth, confirming its oncogenic potential. In EGFR-mutant PC9 NSCLC cells, the same fusion conferred resistance to sunitinib, erlotinib, and osimertinib but sensitized cells to MEK (MAPK/ERK kinase) or RAF (rapidly accelerated fibrosarcoma kinase) inhibitors [146].

While the TMEM87A–RASGRF1 fusion was identified in a patient who had a significant response to sunitinib, the in vitro models and pathway analysis suggest that the fusion itself does not directly confer sensitivity to sunitinib. Instead, it activates the MAPK pathway, which can be targeted by MAPK inhibitors. The fusion may serve as a biomarker for identifying NSCLC patients who could benefit from MAPK inhibitor-based therapies, especially in cases without other known driver mutations.

### 8.3. Mutations

An early study indicated that TMEM132D was commonly mutated in small-cell lung cancer (SCLC) without being influenced by the stage, metastases, or chemotherapy treatment. Thus, it could be involved in SCLC development [147]. The products from their mutated alleles may be potential therapeutic targets in patients.

In a study on pulmonary carcinoid tumors, TMEM41B, TMEM161B, and TMEM155 were identified as significantly mutated genes through a comprehensive genomic analysis that included whole exome sequencing and whole genome sequencing [148]. The mutations were detected using advanced bioinformatics pipelines, including Agilent SureCall and Sentieon TNseq [148]. The significance of these mutations was evaluated using the MutSigCV algorithm, which prioritizes genes based on their mutation frequency and statistical significance. TMEM41B was particularly notable, with a q value of 0.0349, indicating a high level of significance [148]. Furthermore, pathway analysis implicated TMEM41B as being linked to the NF-kB pathway, suggesting its potential role in the deregulation of this pathway in pulmonary carcinoid tumors.

In a Chinese cohort of 182 LUAD, 16 tumors (8.8%) harbored an in-frame three amino acid deletion (Q200del) in the conserved luminal loop of TMEM229A [149]. The alteration was found in lepidic LUAD but absent in micro-papillary LUAD. TMEM229A R76H and M346T mutations were not found as significant genetic alterations in this cohort [149]. In contrast, the TCGA-LUAD cohort showed a much lower mutation rate of 1.0% for TMEM229A mutations (including R76H, M346T, and Q200del) [149]. Further analysis suggested that a lower frequency of the Q200del mutation was significantly associated with positive lymph node metastasis, advanced TNM stages, and positive cancer thrombus [149]. Functionally, overexpression of TMEM229A Q200del inhibited the proliferation and migration of NSCLC cells in vitro [149]. The underlying mechanism involved inactivation of the ERK pathway, as evidenced by reduced levels of phosphorylated ERK and AKT (Ser473), with a more pronounced reduction in p-ERK in the TMEM229A Q200del group compared with the wild-type TMEM229A group [149]. These data position TMEM229A-Q200del as a protective, loss-of-function-like variant.

## 9. Indirect Modulators of Lung Tumors

While the majority of studies have focused on the direct impact of high or low expression of certain TMEMs on tumor progression in lung cancer, as evidenced by validations in cell lines and clinical samples, there are studies that have highlighted the influence of certain TMEMs on the tumor microenvironment of lung cancer, with their effects mediated through extracellular vesicles (EVs), B cells, or endothelial cells rather than directly through lung cancer cells.

### 9.1. TMEM59

TMEM59, also known as DCF1, C1orf8, PRO195, UNQ169, and HSPC001, is a protein that has been implicated in modulation of the tumor microenvironment through EVs. Under hypoxic conditions, A549 lung cancer cells synthesize EVs containing TMEM59, which is associated with enhancing tumor cell stemness. This process is part of a broader response where hypoxic tumors produce hypoxia-sensitive proteins packed into EVs to promote cancer progression, including proliferation, metastasis, angiogenesis, and immune suppression [150].

Aside from being packaged into hypoxia-induced tumor-derived extracellular vesicles, TMEM59 is markedly upregulated during late B-cell differentiation in LUAD, as shown by single-cell RNA-seq and pseudotime analysis, suggesting it may mark mature or activated B cells within the tumor microenvironment [151]. Its presence among 14 ferroptosis-related genes linked to B-cell activation also implies a possible regulatory link among TMEM59, ferroptosis, and antitumor immunity, although the specific functional role of TMEM59 in LUAD remains to be defined [151].

### 9.2. TMEM132A

Accumulating evidence shows that people with LUAD are much more likely to have blood clots in their veins, which is a substantial clinical issue. TMEM132A has been identified as a crosstalk biomarker between LUAD and venous thromboembolism. It is upregulated in LUAD tissue and serum, predicts venous thromboembolism risk, shapes immune cell infiltration, and offers a measurable target for birabresib and abemaciclib intervention [152].

### 9.3. TMEM215

TMEM215, an ER resident two-pass TMEM upregulated by shear stress, safeguards endothelial cells during vessel pruning via downregulation of EZH2 (enhancer of zeste homolog 2) and suppressing BiP (Binding immunoglobulin protein (glucose-regulated protein 78))–BIK (BCL2-interacting killer)–mitochondria Ca^2+^-driven endothelial cell apoptosis. Its downregulation triggers lethal Ca^2+^ influx via mitochondria-associated ER membranes [153]. Although GEPIA shows no significant difference in TMEM215 expression between lung tumors and control tissues, its endothelial pro-survival role positions it as an indirect, druggable modulator of tumor neovascularization.

While TMEM proteins are increasingly recognized as critical regulators of lung cancer pathogenesis, several challenges must be addressed for their clinical translation. First, functional heterogeneity is profound within this family, with some members (e.g., TMEM88) exhibiting dual roles depending on subcellular localization. This demands therapeutic strategies tailored to isoform-specific expression patterns and interactome networks rather than expression levels alone. Second, structural information is lacking for most TMEM proteins, with their subcellular localization and membrane orientation often poorly characterized or unverified. This hampers both the elucidation of their roles and rational drug design. Additionally, many mechanistic links remain correlational rather than causal, with downstream effectors and direct scaffolding functions largely undefined. Closing these gaps requires integrated approaches, including interaction proteomics and genetic rescue experiments with disease-associated variants.

Moreover, the importance of the physiological functions of TMEMs is also understudied, and if they are targeted, then this may raise concerns about potential systemic toxicity due to these proteins’ essential functions in normal tissues. These concerns necessitate tissue-specific delivery systems that distinguish pathological from physiological functions.

Clinical translation also faces hurdles; demethylating agents lack tumor specificity, and RNAi-based therapies are limited by their delivery efficiency. The repurposing of anthelmintics as TMEM16A antagonists highlights the promise of drug repositioning but also underscores the need for rigorous toxicity profiling.

## 10. Conclusions

TMEMs have emerged as important regulators of lung cancer initiation, progression, immune evasion, and drug resistance. Approximately 50 TMEMs have been found to be deregulated and have been studied in lung cancer; some predominantly act as tumor suppressors (e.g., TMEM100 and TMEM196), while others function as oncogenes (e.g., TMEM14A and TMEM158). Their mechanisms span several cancer hallmarks, such as Ca^2+^-channeled proliferation (e.g., ORAI1 and ORAI3), lysosomal exocytosis-driven metastasis (e.g., TMEM106B), EMT (e.g., TMEM52B, TMEM100, TMEM106A, TMEM229A, TMEM139, TMEM158, TMEM160, and TMEM176B), regulation of programed cell death (e.g., TMEM173, TMEM45B, TMEM48, TMEM100, TMEM106A, TMEM164, TMEM166, TMEM176A, TMEM196, TMEM158, and TMEM215), Wnt and β-catenin modulation (e.g., TMEM33 and TMEM100), metabolic rewiring (e.g., TMEM14A and TMEM160), and immune checkpoint reprogramming (e.g., ORAI1 and TMEM184A). The presence of TMEM215 in endothelial cells, TMEM59 in hypoxic tumor-derived extracellular vesicles, and late B cells indicates that TMEM-directed therapies may reshape the tumor microenvironment. Collectively, TMEMs provide a rich, still largely untapped reservoir of prognostic biomarkers, therapeutic targets, and companion diagnostic markers in lung cancer. Their clinical potential is tempered by structural ignorance, mechanistic opacity, and potential systemic toxicity. Future efforts must prioritize structure determination in native lipid environments, functional dissection of context-specific isoforms, and preclinical assessment of therapeutic windows in sophisticated models that recapitulate the tumor microenvironment. Via such balanced, multi-pronged investigations, the translational potential of TMEMs can be more effectively realized.

## Figures and Tables

**Table 1:** TMEM genes with high expression significantly associated with longer survival (PFS or OS) in ≥2 of 4 GEPIA2 analyses and no contradictory trend.

Log-Rank *p* Value ^1^		Raw Values		Normalized by Reference Genes					
				*GAPDH*		*ACTB*		*GNAS*	
Name	Aliases	DFS	OS	DFS	OS	DFS	OS	DFS	OS
*TMEM101*	*—*	/	/	/	M:0.015	/	Q:0.015	/	/
*TMEM108*	*CT124, RTLN*	/	/	/	M:0.039	/	Q:0.031	/	/
*TMEM109*	*SND3, hSND3*	/	/	/	Q:0.0097	Q:2.2 × 10^−5^	Q:0.0095	/	/
*TMEM114*	*—*	/	/	/	Q:0.008	M:0.001	M:0.026	/	/
*TMEM117*	*—*	M:0.00016	/	M:0.0034	M:0.012	M:1 × 10^−4^	/	M:0.0073	/
*TMEM120A*	*NET29, TACAN, TMPIT*	/	/	/	M:0.022	/	M:0.029	/	/
*TMEM123*	*KCT3, PORIMIN, PORMIN*	/	/	/	/	Q:0.0019	/	M:0.049	/
*TMEM130*	*—*	/	M:0.02	/	M:0.0014	/	Q:8.6 × 10^−6^	/	Q:0.024
*TMEM131*	*CC28, PRO1048, RW1, YR-23*	/	/	/	Q:0.045	/	M:0.048	/	/
*TMEM132B*	*—*	/	/	/	Q:0.0032	/	Q:0.021	/	M:0.0093
*TMEM132C*	*PPP1R152*	/	/	/	Q:0.014	Q:0.0017	Q:0.024	/	/
*TMEM132D*	*MOLT, PPP1R153*	/	/	/	Q:0.0076	M:0.017	Q:0.0022	/	/
*TMEM132E*	*DFNB99*	/	/	/	Q:0.021	Q:0.013	Q:0.0012	/	/
*TMEM134*	*—*	/	/	/	Q:0.013	M:0.0017	Q:0.023	/	/
*TMEM138*	*HSPC196*	/	/	/	M:0.00075	M:0.0061	M:0.0025	/	/
*TMEM145*	*—*	/	/	/	Q:0.0019	/	Q:0.019	/	/
*TMEM14A*	*C6orf73, PTD011*	Q:0.014	/	/	Q:0.0014	Q:0.0014	/	M:0.048	/
*TMEM14B*	*—*	/	/	/	Q:0.03	/	M:0.033	/	/
*TMEM150C*	*TTN3*	/	/	/	M:0.0015	/	M:0.025	/	Q:0.037
*TMEM151B*	*TMEM193, C6orf137, bA444E17.5*	/	/	/	M:0.01	Q:0.0065	M:0.0048	/	/
*TMEM154*	*—*	Q:0.019	/	/	/	M:0.00037	/	/	/
*TMEM158*	*BBP, RIS1, p40BBP*	M:0.035	/	M:0.022	M:0.045	M:0.00024	/	M:0.042	/
*TMEM161A*	*AROS-29, AROS29*	/	/	/	M:0.00044	M:0.0023	M:0.016	/	/
*TMEM168*	*—*	/	/	/	M:0.00065	Q:0.018	/	/	Q:0.049
*TMEM17*	*—*	Q:0.023	/	/	M:0.045	Q:0.0028	/	/	/
*TMEM170B*	*—*	/	/	/	Q:0.0049	Q:0.046	M:0.00031	/	/
*TMEM173*	*STING, STING1*	/	/	/	Q:0.0083	/	Q:0.00059	/	Q:0.025
*TMEM174*	*—*	/	/	/	M:0.0097	Q:0.0051	Q:0.028	/	/
*TMEM177*	*—*	/	/	/	M:0.00039	Q:0.015	Q:0.0056	/	/
*TMEM178A*	*TMEM178*	/	/	/	M:0.0028	/	Q:0.0058	/	/
*TMEM178B*	*—*	/	/	/	Q:0.023	M:0.037	M:0.033	/	/
*TMEM179A*	*C14orf90, FLJ42486, TMEM179*	/	/	/	M:0.027	Q:0.014	M:0.029	/	/
*TMEM179B*	*—*	/	/	/	Q:0.022	/	M:0.0098	/	/
*TMEM180*	*SLC68A1, MFSD13A, FLJ22529, C10orf77, bA18I14.8*	/	/	/	M:0.021	/	Q:0.028	/	/
*TMEM185A*	*CXorf13, FAM11A, FRAXF, ee3*	/	/	/	Q:0.015	/	M:0.00037	/	/
*TMEM186*	*C16orf51*	/	/	/	Q:0.0029	Q:0.038	M:0.029	/	/
*TMEM196*	*—*	/	/	/	M:0.0081	Q:0.0038	M:0.04	/	/
*TMEM198*	*TMEM198A*	/	/	/	Q:0.008	Q:0.025	Q:0.015	/	/
*TMEM200C*	*TTMA*	/	/	/	Q:0.0034	Q:0.033	M:0.013	/	/
*TMEM201*	*Ima1, NET5, SAMP1*	M:0.0025	/	/	Q:0.011	Q:5.8 × 10^−5^	/	/	/
*TMEM202*	*—*	/	/	/	M:0.0027	Q:0.0029	M:0.045	/	/
*TMEM203*	*HBEBP1*	/	/	/	Q:0.0042	/	M:0.002	/	/
*TMEM207*	*UNQ846*	/	/	/	M:0.0025	Q:0.0031	Q:0.019	/	/
*TMEM209*	*NET31*	/	/	/	M:0.0095	Q:0.0056	M:0.01	/	/
*TMEM210*	*—*	/	/	/	M:0.0075	Q:0.011	Q:0.034	/	/
*TMEM211*	*LHFPL7*	M: 0.024	/	/	Q:0.0059	M:0.0016	M:0.025	/	/
*TMEM212*	*—*	/	/	/	Q:0.027	M: 0.006	M:0.008	/	/
*TMEM213*	*—*	/	/	/	M:0.0065	Q:0.019	Q:0.00084	/	/
*TMEM215*	*—*	/	/	/	M:0.002	Q:0.0014	Q:0.0012	/	M:0.033
*TMEM216*	*HSPC244, RP98*	/	/	/	Q:0.00034	Q:0.0031	M:0.011	/	/
*TMEM218*	*JBTS39*	M:0.022	/	/	/	Q:0.0036	/	/	/
*TMEM221*	*Jiraiya*	/	/	/	Q:0.0096	M:0.0094	M:0.042	/	/
*TMEM225*	*PMP22CD, SPATA47, PPP1R154*	/	/	/	M:0.0085	Q:0.0076	M:0.026	/	/
*TMEM227*	*TPRA1, GPR175, TPRA40*	M:0.035	/	/	Q:0.011	Q:0.00025	Q:0.045	/	/
*TMEM229A*	*—*	/	/	/	M:0.0051	Q:0.015	Q:0.008	/	/
*TMEM229B*	*C14orf83*	/	/	/	M:0.011	/	Q:0.03	/	/
*TMEM235*	*ARGM1*	/	/	/	M:0.0019	Q:0.0031	M:0.016	/	/
*TMEM236*	*FAM23A/B, bA162I21.2, bA16O1.2*	/	/	/	M:0.037	Q:0.015	Q:0.0015	/	/
*TMEM237*	*ALS2CR4, JBTS14*	M:0.016	/	/	Q:0.023	Q:0.00022	/	M:0.045	/
*TMEM238*	*—*	/	/	/	M:0.0073	/	Q:0.015	/	M:0.035
*TMEM241*	*SLC35D4, C18orf45, hVVT*	/	/	/	Q:0.00092	/	M:0.00066	/	/
*TMEM242*	*BM033, C6orf35*	/	/	/	M:0.018	Q:0.0056	Q:0.039	/	/
*TMEM243*	*C7orf23, MM-TRAG, MMTRAG*	/	Q:0.028	/	Q:0.0015	/	M:2.2 × 10^−6^	/	Q:0.044
*TMEM244*	*C6orf191, bA174C7.4*	/	/	/	M:0.0011	Q:0.0061	M:0.031	/	/
*TMEM246*	*C9orf125, PGAP4*	Q: 0.00039	/	Q:0.00017	/	Q:0.00011	/	Q:0.00083	/
*TMEM247*	*—*	/	/	/	M:0.0022	Q:0.0022	Q:0.023	/	/
*TMEM25*	*—*	/	M:0.02	/	Q:0.0012	M:0.022	Q:0.0069	/	/
*TMEM251*	*LYSET, DMAN, GCAF, C14orf109*	/	/	/	M:8.1 × 10^−5^	/	M:0.001	/	Q:0.017
*TMEM252*	*C9orf71*	/	/	/	M/Q:0.013	Q:0.0077	Q:0.0011	/	/
*TMEM26*	*—*	/	/	/	Q:0.023	Q:0.011	M:0.046	/	/
*TMEM260*	*C14orf101, SHDRA*	/	/	/	Q:0.00026	Q:0.026	Q:0.0076	/	M:0.017
*TMEM261*	*DMAC1, C9orf123, MGC4730*	/	/	/	Q:0.0044	M:0.015	Q:0.011	/	/
*TMEM31*	*—*	/	/	/	Q:0.017	Q:0.048	M:0.03	/	/
*TMEM35*	*TMEM35A, NACHO, TUF-1*	/	Q:0.013	/	Q:0.0027	Q:0.031	Q:0.00067	/	Q:0.011
*TMEM38A*	*TRIC-A, TRICA*	/	Q:0.0053	/	Q:6 × 10^−4^	/	M:0.0039	/	Q:0.017
*TMEM40*	*—*	M:1.5 × 10^−5^	/	M:2.3 × 10^−5^	/	M:3.9 × 10^−7^	/	M:5.5 × 10^−5^	/
*TMEM42*	*—*	/	/	/	M:0.0037	/	Q:0.01	/	/
*TMEM50B*	*C21orf4, HCVP7TP3*	/	Q:0.014	/	Q:0.0046	/	Q:4.7 × 10^−5^	/	/
*TMEM52*	*—*	/	/	/	Q:0.0039	M:0.022	M:0.0084	/	M:0.03
*TMEM55B*	*PIP4P1, C14orf9, MGC26684*	/	/	/	Q: 0.012	/	Q:0.0011	/	/
*TMEM57*	*MACO1, MACOILIN, FLJ10747*	/	/	/	M:0.014	M:0.029	M:0.00097	/	M:0.013
*TMEM60*	*C7orf35, DC32*	/	/	/	M:0.0029	/	Q:0.00011	/	Q:0.014
*TMEM63C*	*C14orf171, CSC1, SPG87*	/	/	/	Q:0.00064	M:0.021	Q:0.0026	/	M:0.011
*TMEM69*	*C1orf154*	/	/	/	Q:0.0012	Q:0.003	Q:0.026	/	/
*TMEM72*	*C10orf127, KSP37*	/	/	/	M:0.0051	Q:0.0097	Q:0.0062	/	/
*TMEM82*	*—*	/	/	/	Q:0.014	/	Q:0.023	/	/
*TMEM8B*	*C9orf127, FP588, LINC00950, NAG-5, NAG5, NGX6, NGX6a*	/	/	/	M:0.0082	Q:0.0064	Q:0.035	/	/
*TMEM8C*	*MYMK, TMEM226, MYOMAKER*	Error	Error	/	M:0.02	Q:0.022	Q:0.031	/	/
*TMEM94*	*ERMA, IDDCDF, KIAA0195*	/	/	/	Q:0.032	/	Q:0.046	/	/
*TMEM95*	*UNQ9390*	/	/	/	M:0.0028	Q:0.007	M:0.029	/	/

^1^ “/” indicates *p* ≥ 0.05 (not statistically significant); M indicates a *p* value calculated using the median cut-off point; and Q indicates a *p* value calculated using the quartile cut-off point. For each gene, the cut-off that produced the smaller log-rank *p* value is presented. PFS = progression-free survival; OS = overall survival.

**Table 2:** TMEM genes with low expression significantly associated with longer survival (PFS or OS) in ≥2 of 4 GEPIA2 analyses and no contradictory trend.

Log-Rank *p* Value ^1^		Raw Values		Normalized by Reference Genes					
				*GAPDH*		*ACTB*		*GNAS*	
Name	Aliases	DFS	OS	DFS	OS	DFS	OS	DFS	OS
*TMEM106A*	*—*	M:0.0019	/	/	/	/	/	M:0.0063	Q:0.048
*TMEM126B*	*HT007, MC1DN29*	M:0.031	Q:0.029	Q:0.031	/	/	/	M:0.017	/
*TMEM139*	*—*	Q:0.014	/	Q:0.0029	/	/	/	Q:0.025	/
*TMEM141*	*—*	Q:0.03	Q:0.034	M:0.042	/	/	/	Q:0.024	/
*TMEM144*	*SLC35G7*	M:0.00062	M:0.035	M:0.05	/	/	/	Q:0.019	/
*TMEM165*	*CDG2K, FT27, GDT1, SLC64A1, TMPT27, TPARL*	Q:0.0016	/	Q:0.019	/	/	/	M:0.03	/
*TMEM167A*	*TMEM167, kish*	M:0.0057	Q:0.026	/	/	/	/	Q:0.046	/
*TMEM184A*	*SDMG1, SLC51C1*	M:0.028	/	/	/	/	/	Q:0.034	/
*TMEM2*	*CEMIP2*	Q:0.00027	/	Q:0.028	/	/	/	M:6.6 × 10^−5^	/
*TMEM208*	*HSPC171, SND2, hSND2*	M:0.0024	M:0.014	M:0.027	/	/	/	/	/
*TMEM214*	*—*	Q:0.0036	/	Q:0.018	/	/	/	Q:0.0081	/
*TMEM217*	*C6orf128, dJ355M6.2*	Q:0.014	/	M:0.028	/	/	/	Q:0.014	/
*TMEM220*	*—*	Q:0.0085	Q:0.03	M:0.027	/	/	/	Q:0.026	/
*TMEM255B*	*FAM70B*	M:0.033	/	Q:0.032	/	/	/	/	/
*TMEM263*	*C12orf23*	Q:0.03	/	M:0.014	/	/	/	Q:0.002	/
*TMEM265*	*IFITMD8*	Q:0.033	/	Q:0.05	/	/	/	/	/
*TMEM45A*	*DERP7, DNAPTP4*	/	Q:0.03	/	/	/	/	/	M:0.022
*TMEM45B*	*—*	M:0.0013	/	M:0.033	/	M:0.016	/	M:5 × 10^−4^	/
*TMEM51*	*C1orf72*	M: 0.00018	M: 0.0055	Q:0.0016	/	/	/	Q:0.0041	/
*TMEM61*	*—*	M:1.5 × 10^−5^	/	M:0.037	/	M:0.025	/	M:0.00095	/
*TMEM6*	*PGAP6, M83, TMEM8, TMEM8A, GPI-PLA2*	Q:2.3 × 10^−5^	/	M:0.027	/	M:0.029	/	Q:0.00019	/
*TMEM81*	*HC3107, KVLA2788, UNQ2788*	Q:0.0017	Q:0.034	M:0.027	/	/	/	M:0.036	/
*TMEM87B*	*—*	Q:0.023	/	Q:0.036	/	/	/	Q:0.045	/
*TMEM92*	*—*	M:0.00039	Q:0.0066	M:0.0012	/	Q:0.034	/	M:0.00047	/

^1^ “/” indicates *p* ≥ 0.05 (not statistically significant); M indicates a *p* value calculated using the median cut-off point; and Q indicates a *p* value calculated using the quartile cut-off point. For each gene, the cut-off that produced the smaller log-rank *p* value is presented.

**Table 3:** Summary of TMEM proteins with tumor-suppressive roles in lung cancer.

TMEM ID	Clinical Association	Function or Role	Molecular Mechanism	References
TMEM8B-a		Tumor metastasis suppressor	Ubiquitination-independent, ezrin-mediated proteasomal degradation	[87]
TMEM17	Protein expression: lung cancer < adjacent normal tissue Expression↓ → differentiation↓; TNM stage and lymph node metastasis↑; OS↓		**Downstream effectors:** TMEM17↑ → p-ERK/p90RSK/Snail↓; Occludin/ZO-1↑ → invasion and migration↓	[88]
TMEM100	Protein expression: NSCLC < adjacent normal lung tissues; mainly expressed in the cytomembrane Expression↓ → OS↓	**Overexpression in NSCLC cell lines:** proliferation↓ in vitro and in vivo; migration and invasion↓	TMEM100 worked as a cancer suppressor gene mainly by inhibiting the **TNF signaling pathway**	[89]
	mRNA and protein expression: noninvasive or invasive lesions of early-stage LUAD < normal tissue Expression↑ → relapse-free survival↑			[90]
	mRNA expression (TCGA): NSCLC < normal tissues Expression↑ → TNM stage↓; better prognosis	**Overexpression in NSCLC cell lines:** apoptosis and autophagy↑; tumor growth↓	**Downstream effectors:** • BAX/BCL2 → apoptosis; • PI3K/AKT signaling↓ → autophagy Autophagy↓ → TMEM100-induced apoptosis↑ (compensate for the cell death)	[91]
	mRNA expression: NSCLC < paired peritumoral tissues (GEO) mRNA expression↓ → OS↓ (GEO)	**Overexpression in NSCLC cell lines:** colony formation↓; apoptosis↑	**Upstream regulator:** miR-106b → TMEM100↓ **Downstream effectors:** TMEM100 → survivin↓/Bim/caspase-3	[92]
	mRNA expression: NSCLC < normal tissues Expression↑ → OS↑	**Overexpression in NSCLC cell lines:** proliferation↓, migration↓, invasion↓, and TGF-β1-induced EMT↓ in vitro; metastasis↓ in vivo	**Upstream regulator:** HDAC6 → TMEM100↓ **Downstream effectors:** TMEM100 → Wnt/β-catenin pathway↓	[93]
	mRNA expression: LUAD < non-tumor tissue	**Overexpression in LUAD cell lines:** migration and invasion↓	**Upstream regulator:** circ_0000567 acts as a sponge for miR-421 and prevents miR-421 from degrading TMEM100 mRNA	[94]
TMEM106A	mRNA expression: NSCLC < adjacent normal lung tissues	**Overexpression in NSCLC cell lines:** apoptosis↑; proliferation↓, migration↓, invasion↓, and EMT↓	**Downstream effectors:** TMEM106A → PI3K/Akt/NF-κB activation↓	[95]
TMEM139	mRNA and protein expression: NSCLC < adjacent normal lung tissues Expression↑ → OS↑ and DFS↑	**Overexpression in NSCLC cell lines:** migration↓, invasion↓, and EMT↓ in vitro; metastasis↓ in vivo	TMEM139 binds E-cadherin at the plasma membrane and focal adhesion sites and prevents the lysosomal degradation of E-cadherin	[96]
TMEM164	mRNA and protein expression: LUAD< normal lung tissues (PHA database and clinical samples) Expression↑ in LUAD → better prognosis	**mRNA expression:** A549 and NCI-H358 < Base-2b **Overexpression in LUAD cell lines:** proliferation↓, migration↓, invasion↓, and autophagy↑ in vitro; synergizes with anti-PD-1 in vivo	**Downstream effectors:** TMEM164↑ → ATG5-dependent autophagosome formation (autophagy) → ferroptosis mainly through GPX4 degradation	[97]
		**Downregulation:** lipid peroxidation and autophagy-dependent ferroptosis↓; cell viability↑ in A549	**Upstream regulator:** TRIM59 → ubiquitination and degradation of TMEM164	[98]
TMEM176A	Methylated in 53.66% of primary lung cancer	**Overexpression:** colony formation↓, cell proliferation↓, migration↓, and invasion↓ in vitro; apoptosis↑ and G2/M phase arrest↑ in vitro; H1299 cell xenograft growth↓ in vivo	**Downstream effectors:***TMEM176A* methylation → activation of ERK signaling↑ but not ATM or ATR pathway	[99]
TMEM196	mRNA and protein expression: lung cancer < adjacent normal tissues Expression↑ in LUAD and LUSC → better prognosis only in TNM stages I–II, not III–IV	**Overexpression:** migration and invasion↓ in vitro; lung and liver metastasis↓ in vivo **Downregulation:** migration and invasion↑ in vitro **Lung metastases produced by tail vein-injected B16 cells:** tumor volume: KO-TMEM196 > wild-type mice; number of nodules: KO-TMEM196 > wild-type mice	**Downstream effectors:** TMEM196↑ → Wnt signaling pathway↓ and β-catenin promoter transcription↓	[100]
	Protein expression: lung cancer < adjacent normal tissues; Protein expression↑ in LUAD/LUSC/TNM stages I–II or III–IV → better prognosis; Frequency of methylation: Positively correlates with pathological severity (no methylation in normal lung tissues); Lung cancer > adjacent normal tissues; Hypermethylation → poor differentiation and advanced stage	A549, SPC-A-1, 95D, H1975, H358, H1650, LTEP-a-2, H1395, H446 and H460 cell lines: hypermethylated; HBE cell line: unmethylated **Overexpression:** apoptosis↑; S phase↓; G2 or M phase arrest↑; proliferation↓, clonogenicity↓, and migration↓ in vitro; tumor formation↓ in vivo; **Downregulation:** opposite effects	**TMEM196 methylation negatively associated with expression level** during chemically induced rat lung carcinogenesis in cell lines and in clinical samples **Downstream effectors:** TMEM196↑ → p21↑ and Bax↑; cyclin D1↓, c-Myc↓, CD44↓, and β-catenin↓	[101]
	Methylation↑ or expression↓ is an independent prognostic marker for poorer survival (TCGA); Methylation levels in plasma or sputum correlate with corresponding paired tissue and effectively discriminate patients from healthy subjects			[102]
TMEM213	mRNA expression↑ in LUAD (especially in adjuvant paclitaxel-carboplatin treated patients) → 3-year OS rate↑ and OS↑; an independent predictor for OS (TCGA)		**Related pathway:** drug metabolism cytochrome P450, ABC transporter, butyric acid, arachidonic acid, tryptophan, fatty acid, histidine, and bile acid synthesis and other metabolic pathways (TCGA GSEA database, KEGG gene sets)	[84]
TMEM229A	mRNA and protein expression: NSCLC < adjacent normal lung tissues Protein expression↓ → TNM stage↑, cancer thrombus↑, differentiation↓, and lymph node metastasis↑ Expression↑ in LUAD/LUSC: OS↑ (GEO, EGA, and TCGA)	Expression: A549, H23, 95D, H226, and H1975 cell lines < BEAS-2B cells **Overexpression:** proliferation↓, colony formation, EMT↓, migration↓, and invasion↓ Knockdown exerted opposite effects	**Downstream effectors:** TMEM229A↑ → p-ERK/p-AKT↓ (knockdown exerted opposite effects), which was partially reversed by ERK inhibitor PD98059	[86]
TMEM245	mRNA expression↑ in NSCLC patients → OS↑			[85]
TMEM88	mRNA expression: LUAD < adjacent normal tissues; mRNA expression↑ → OS↑	miR-708 expression↑ → TMEM88↓ → cell proliferation, invasion, and migration↑	**Upstream Regulator:** miR-708 binds and reduces the transcript for TMEM88	[103]
	Hypermethylation: NSCLC > adjacent normal tissues; hypermethylation in NSCLC → tumor size↑, mRNA expression↓, and OS↓ mRNA expression: NSCLC < adjacent normal tissues; not associated with OS	**Demethylation agent treated A549 and H1299:** TMEM88 expression↑, proliferation↓, migration and invasion↓ (abolished by TMEM88 siRNA), arrested cell cycle at the G2 or M phase↑	**TCGA data:** weak positive correlations with Wnt pathway factor DVL1, FZD4/5, and ROR1	[104]

“↑” = increase; “↓” = decrease; “→” = the direction of the pathway; ATG5 = autophagy-related 5; ATM = Ataxia telangiectasia mutated; ATR = AT- and Rad3-related; BAX = BCL2-associated X protein; BCL2 = B-cell lymphoma 2; DFS or DSS = disease-free or -specific survival; DVL = disheveled segment polarity protein; EGA = European Genome-phenome Archive; EMT = epithelial-mesenchymal transition; ERK = extracellular signal-regulated kinase; FZD = frizzled class receptor; GEO = Gene Expression Omnibus database; GPX4 = glutathione peroxidase 4; HDAC6 = histone deacetylase 6; KO = knockout; LUAD = lung adenocarcinoma; LUSC = lung squamous cell carcinoma; miR = microRNA; NSCLC = non-small cell lung carcinoma; PD-1 = programmed cell death protein 1; PI3K/Akt/NF-κB = phosphoinositide 3-kinase, protein kinase B, and nuclear factor-κB; ROR1 = receptor tyrosine kinase-like orphan receptor 1; TCGA = The Cancer Genome Atlas database; TMEM = transmembrane protein; TNF = tumor necrosis factor; TNM = tumor, node, and metastases; Wnt = wingless-type MMTV integration site family.

**Table 4:** Summary of TMEM proteins associated with tumor promotion in lung cancer.

TMEM ID	Clinical Association	Function or Role	Molecular Mechanism	References
TMEM14A		TMEM14A↓ (via circ_0003645↓) → NSCLC proliferation, migration, invasion↓	**Upstream regulator:** circ_0003645 sponges miR-1179 → TMEM14A mRNA stabilization	[113]
	mRNA expression: LUAD > normal tissues (GEPIA2) LUAD: TMEM14A↑ → OS↓	**Downregulation:** NSCLC proliferation↓ and ATP↓	**Upstream regulator:** AXL → TMEM14A↑ **Downstream effectors:** TMEM14A↑ → mitochondrial ATP↑ → proliferation↑	[114]
TMEM33	mRNA and protein expression: LUAD > matched non-carcinoma samples TMEM33↑ in LUAD: tumor grade (GEPIA)↑; lymph node metastasis↑; TNM stage↑; OS↓	**Expression:** LUAD cell lines > BEAS-2B cells **Downregulation:** proliferation↓, invasion↓, and stemness↓ in vitro; tumor growth and metastasis↓ in vivo	**Upstream regulator:** miR-214-3p → TMEM33↓ **Downstream effectors:** TMEM33↓ → Wnt and β-catenin (β-cat, c-Myc, Cyclin D1)↓	[115]
TMEM45A	Protein expression and prevalence: primary LUAD > nontumorous tissues	**mRNA expression**: late stage in vivo > early stage in vivo > in vitro 2D or 3D culture [116]		[117]
TMEM45B	mRNA expression↑ in lung cancer → OS↓	**Downregulation:** proliferation, migration, and invasion↓ in vitro; tumor growth↓ in vivo; cell cycle arrest↑ (reduction of G1/S transition) and apoptosis↑	**Downstream effectors:** cell cycle-related proteins (CDK2, CDC25A, and PCNA), cell apoptosis-related proteins (Bcl-2, Bax, and cleaved caspase 3), and metastasis-related proteins (MMP-9, Twist, and Snail)	[118]
TMEM74	Protein expression: LUAD and LUSC > adjacent normal tissues; protein expression↑ in LUAD and LUSC → OS↓	**Expression:** lung cancer cell lines > normal cell lines **Overexpression:** proliferation↑ **Downregulation:** proliferation↓	Reported as an autophagy inducer, promoting tumor cell proliferation by triggering autophagy	[119]
TMEM88 isoform 1 (CRA-a)	Protein expression: NSCLC (cytosol, nuclear, and membrane) > paired noncancerous tissues (membrane/cytosol) Cytosolic expression↑ in NSCLC: OS↓; histologic differentiation, lymph node metastasis, and TNM stage CRA-a protein expression: NSCLC tissues > paired noncancerous tissues; CRA-b: NSCLC tissues ≈ paired noncancerous tissues	**Endogenous CRA-a expression:** A549, H1299, H292, PG-BE1, PG-LH7, SPC-A-1, and LTEP-A-2 cells > HEB cells > LK2 and H460 cells **CRA-a subcellular localization:** cytoplasm and nuclear (except LK2 cells: membrane) **Exogenous overexpression:** Membrane-localized CRA-a (LK2 cells): colony formation↓, proliferation↓, migration, and invasion↓ in vitro; tumor volume↓, and metastasis↓ in vivo. Cytosol-localized CRA-a (H1299, A549, and SPC-A-1 cells): migration and invasion↑ in vitro; metastasis↑ in vivo. Verified by CRA-a downregulation.	**Membrane-localized CRA-a** → Wnt signaling↓ **Downstream effectors:** Cytosol-localized CRA-a↑ (H1299, A549) → active form of p38↑ (phosphorylated-P38) → GSK3β (Thr390)↑, ATF2↑, and Snail↑; tight junction-associated proteins ZO-1↓, and occludin↓. CRA-a (H1299, SPC-A-1)↓ → Snail expression↓, occludin↑, and ZO-1↑.	[120]
TMEM98			**Upstream Regulator:** miR-29c-5p	[121]
	mRNA expression: lung carcinoma tissues > adjacent normal tissues	**Downregulation (siRNA):** proliferation↓, migration↓, and invasion↓ in NSCLC cell lines	**Downstream effectors:** MMP-2, MMP-9, RhoC and MTA1	[122]
TMEM106B	LUAD patients (19%) with elevated gene amplification/mRNA expression of TMEM106B → DFS↓ and OS↓ (TCGA)	**Overexpression** in nonmetastatic 393P cells: metastasis↑; no influence on primary tumor growth **Downregulation** in metastastic 344SQ cells: metastasis↓; no influence on primary tumor growth Conditioned medium from 344SQ/393P cells (TMEM106B↑) → 393P parental cell metastasis↑ TMEM106B-mediated cancer cell invasion and metastasis in vivo	**Downstream effectors:** TMEM106B↑ → nuclear translocation of TFEB↑ → expression of lysosomal genes of CLEAR pathway↑ → formation of enlarged vesicular lysosomes containing high levels of active cathepsins↑ → calcium-dependent exocytosis of lysosomes↑ → releasing active lysosomal proteases↑ in ECM → cancer cell invasion and metastasis↑	[123]
TMEM116	Protein expression: NSCLC > non-tumor areas mRNA expression↑ → OS↓(GEPIA)	**Downregulation**: proliferation↓, migration↓, and invasion↓ in vitro and metastasis↓ in tail vein injection mice model	**Downstream effectors:** TMEM116↓ → PDPK1/AKT/FOXO3A signaling pathway↓ → accumulation of TAp63	[124]
TMEM158		**Downregulation** in PC-9 and CDDP cells; reduction in chemo-resistance against cisplatin		[125]
	mRNA expression: LUAD > healthy tissues (TCGA-LUAD and GSE140797; verified at RNA and protein levels using clinical samples); LUAD patients without lymph node metastasis < patients developed lymph node metastasis. Duration of smoking history↑ → TMEM158 expression↑ in LUAD → OS↓ and DSS↓.	mRNA and protein expression: A549, PC9, and H1650 > non-cancerous BEAS-2B cell line **Overexpression:** proliferation↑, cell cycle↑ (cells in G2/M phase), colony formation↑, migration↑, and invasion↑; cells arrested at the G0/G1 phase↓, and apoptosis↓ **Downregulation**: opposite effects	Co-expression: external encapsulating construction organization, collagen-containing ECM, and ECM structural constituent-related genes **Downstream effectors:** TMEM158↑ → TWIST1↑ and physical interaction with TWIST1 → activates PI3K/AKT signaling	[126]
	Expression↑ → stage↑, OS↓	**Overexpression in lung cancer:** EMT↑, migration↑ **Downregulation:** opposite effects	**Upstream regulator:** hypoxia via HIF-1α → TMEM158↑ **Downstream:** TMEM158 links to EMT, hypoxia, and tumor-promoting pathways	[127]
TMEM160	Protein expression: LUAD > adjacent non-tumor tissue; Predominantly localized in cytoplasm in LUAD	**Subcellular localization cycles with mitosis of lung cancer cells:** interphase, primarily in nucleus; pro-metaphase-anaphase-telophase, predominantly in cytoplasm; cytokinesis; returns to chromosomes while remaining cytoplasmic distribution Protein expression: A549 > BEAS cells. TMEM160↓ (A549) → proliferation↓, migratory ability↓ in vitro; tumor volume↓, necrotic areas↓ in vivo	TMEM160 interactome enriched in: apical junctions, xenobiotic metabolism, glycolysis, EMT, ROS, UV response DNA, the P53 pathway, and the mitotic spindle, especially nucleocytoplasmic transport, with nucleoporin NUP50, importin KPNA6, and SRRM1 (Co-IP-MS GSEA); DNA replication, amino acid biosynthesis, and cell cycle (UALCAN database)	[128]
TMEM176B	mRNA and protein expression: LUAD > adjacent normal tissues> LUSC; stages II and III > stage I (TCGA/GTEx and tissue arrays) mRNA expression↑ in LUAD → OS↓ (GEPIA2)	**Overexpression in LUAD cell lines (PC9 and A549):** cell proliferation↑, invasion↑, migration↑, cell–matrix adhesion↑ in vitro; tumor growth↑ in vivo **FGFR inhibitors (fexagratinib and infigratinib) and JNK inhibitor (SP600125) but no ERK, p38, AKT, or PI3K inhibitor treatment:** the enhancements↓ caused by TMEM176B overexpression in vitro	**Downstream effectors:** TMEM176B↑ → ligand–receptor interactions (primarily involved in JAM, SPP1, gelatin, and ECMs, including fibronectin and collagen) → interaction strength between cancer cells and endothelial cells↑ TMEM176B↑ → FGFR1/JNK/vimentin/Snail axis and E-cadherin↓ → EMT↑	[129]
TMEM209	Widely expressed in lung cancer; mRNA expression: lung cancer > normal lung Localization: nuclear envelope, Golgi apparatus, and cytoplasm	**Overexpression**: COS-7 and SBC-3 cell growth↑; **Downregulation**: opposite effects	**Downstream effectors:** TMEM209 interacts with nucleoporin protein NUP205 (MS) → NUP205 stabilization↑ and c-Myc in the nucleus↑	[130]
TMEM243		mRNA expression: sensitive < low acquired paclitaxel-resistant < high acquired paclitaxel-resistant NCI-H446 cells. **Downregulation:** resensitize resistant cells to paclitaxel, proliferation↓ and invasion↓	**Upstream regulator:** A2M↑ → TMEM243↓ → resensitize the resistant cells to paclitaxel	[131]

“↑” = increase; “↓” = decrease; “→” = the direction of the pathway; A2M = alpha-2-macroglobulin; AXL = AXL receptor tyrosine kinase; CDK2 = Cyclin-dependent kinase 2; CLEAR = coordinated lysosomal expression and regulation; Co-IP = co-immunoprecipitation; ECM = extracellular matrix; FGFR1 = fibroblast growth factor receptor 1; FOXO3A = forkhead box O3; JAM = junctional adhesion molecule; JNK = c-Jun N-terminal kinase; HIF-1α = hypoxia-inducible factor 1-alpha; GSEA = gene set enrichment analysis; KPNA6 = karyopherin subunit alpha 6; MS = mass spectrometry; NUP = nucleoporin; PDPK1 = 3-phosphoinositide-dependent protein kinase 1; RhoC = Ras homolog family member C;ROS = reactive oxygen species; SPP1 = secreted phosphoprotein 1 (osteopontin); TAp63 = tumor protein p63, transcriptionally active isoform; TFEB = transcription factor EB; TWIST1 = Twist family bHLH transcription factor 1;ZO-1 = Zonula occludens-1.

**Table 5:** Summary of TMEM proteins listed in Section 3, Section 4, Section 5 and Section 6.

Article Section	Name	Log-Rank *p* Value ^1^					
		LUAD + LUSC		LUAD		LUSC	
		DFS	OS	DFS	OS	DFS	OS
Section 3	TMEM8B	/	/	/	(+)Q:0.033	/	/
	TMEM17	(+)Q:0.023	/	(−)M:0.042	(−)Q:0.019	/	/
	TMEM52B	(−)Q:0.049	/	/	/	(−)Q:0.027	/
	TMEM100	/	/	/	/	/	/
	TMEM106A	(−)M:0.0019	/	/	/	(−)M:0.047	(−)Q:0.02
	TMEM139	(−)Q:0.014	/	/	/	/	/
	TMEM164	/	/	/	/	(−)M:0.03	/
	TMEM176A	/	/	/	/	/	/
	TMEM196	/	/	/	/	Error	Error
	TMEM213	/	/	(+)M:0.0034	(+)Q:0.0083	/	/
	TMEM229A	/	/	/	/	Error	Error
	TMEM245	/	/	/	/	(−)M:0.012	/
Section 4	TMEM14A	(+)Q:0.014	/	(−)Q:0.046	(−)Q:0.0058	(+)Q:0.026	(+)Q:0.027
	TMEM33	/	(−)Q:0.011	/	(−)Q:0.017	/	/
	TMEM45A	/	(−)Q:0.03	/	(−)Q:0.0018	/	/
	TMEM45B	(−)M:0.0013	/	/	/	/	/
	TMEM74	(−)Q:0.041	/	/	/	/	/
	TMEM98	(−)M:0.037	/	/	/	/	/
	TMEM106B	/	/	(−)M:0.017	(−)M:0.0051	/	/
	TMEM116	/	/	/	/	/	/
	TMEM158	(+)M:0.035	/	/	(−)M:0.023	/	/
	TMEM160	/	/	/	/	/	/
	TMEM176B	/	/	/	/	/	/
	TMEM179A	/	/	/	/	/	/
	TMEM209	/	/	/	/	/	/
	TMEM243	/	(+)Q:0.028	(+)M:0.0099	(+)M:0.015	/	/
Section 5	TMEM88	/	/	/	/	/	(−)Q:0.0075
Section 6	TMEM92	(−)M:0.00039	(−)Q:0.0066	/	/	(−)M:0.042	(−)Q:0.0029
	TMEM161A	/	/	/	/	/	/
	TMEM163	/	(+)Q:0.017	/	(+)Q:0.0049	/	/
	TMEM184A	(−)M:0.028	/	/	/	/	/
	TMEM125	(−)M:0.018	/	(+)M:0.024	(+)M:9.1 × 10^−5^	/	(−)M:0.043
	TMEM164	/	/	/	/	(−)M:0.03	/

^1^ “/” indicates *p* ≥ 0.05 (not statistically significant); M indicates a *p* value calculated using the median cut-off point; and Q indicates a *p* value calculated using the quartile cut-off point. For each gene, the cut-off that produced the smaller log-rank *p* value is presented. Directions in parentheses: “+” indicates positive correlation between high expression and longer survival; “−“ indicates negative correlation.

## Data Availability

No new data were created or analyzed in this study.

## Abbreviations

The following abbreviations are used in this manuscript:

TMEM	transmembrane
EGFR	epidermal growth factor receptor
ALK	anaplastic lymphoma kinase
ICB	immune checkpoint blockade therapy
ER	endoplasmic reticulum
GPCRs	G protein-coupled receptors
STING1	stimulator of interferon response cGAMP interactor 1
IFN-I	type-I IFN
ANO1	anoctamin-1
ORAI1	ORAI calcium release-activated calcium modulator 1
Wnt	Wingless-type MMTV integration site family
CRAC	calcium release-activated calcium
STIM1	stromal interaction molecules 1
SOCE	store operated Ca^2+^ entry
ARC	arachidonic acid-regulated Ca^2+^
NSCLC	non-small cell lung cancer
LUAD	lung adenocarcinoma
CDK	Cyclin-dependent kinase
miR	microRNA
NDC1	NDC1 transmembrane nucleoporin
CEMIP	cell migration-inducing and hyaluronan-binding protein
VMP1	Vacuole membrane protein 1
DRAM2	DNA damage regulated autophagy modulator 2
EMC6	ER membrane protein complex subunit 6
EVA1A	eva-1 homolog A
PEDS1	plasmanylethanolamine desaturase-1
IGFLR1	IGF-like family receptor 1
RNFT2	ring finger protein, transmembrane 2
WDR82	WD repeat domain 82
PFS	progression-free survival
OS	overall survival
GEPIA	Gene Expression Profiling Interactive Analysis
GEO	Gene Expression Omnibus database
TCGA	The Cancer Genome Atlas
EGA	European Genome-phenome Archive
ATM	Ataxia telangiectasia mutated
ATR	AT- and Rad3-related
DFS/DSS	disease-free or specific survival
DVL	disheveled segment polarity protein
Snail	Snail family transcriptional repressor 1 (used interchangeably with SNAI1)
ZO-1	Zonula occludens-1
PDPK1	3-phosphoinositide-dependent protein kinase 1 For clarity, we use the official gene symbol PDPK1 throughout this review (formerly called PDK1 in many original studies)
TWIST1	twist family bHLH transcription factor 1
FOXO3A	Forkhead box O3
BAX	BCL2-associated X protein
BCL2	B-cell lymphoma 2
HIF-1α	hypoxia-inducible factor 1-alpha
EMT	epithelial-mesenchymal transition
FZD	frizzled class receptor
GPX4	glutathione peroxidase 4
HDAC6	histone deacetylase 6
KO	knockout
LUSC	lung squamous cell carcinoma
PI3K/Akt/NF-κB	phosphoinositide 3-kinase, protein kinase B, and nuclear factor-κB
ERK	extracellular signal-regulated kinase
ROR1	receptor tyrosine kinase like orphan receptor 1
SPP1	secreted phosphoprotein 1 (osteopontin)
TAp63	tumor protein p63, transcriptionally active isoform
aNL	adjacent histologically normal lung
TNM	TNM stage
IHC	Immunohistochemistry
HBE1	human bronchial epithelial cell line
HPA	Human Protein Atlas
MS4A	membrane-spanning 4A
A2M	alpha-2-macroglobulin
AXL	AXL receptor tyrosine kinase
RhoC	Ras homolog family member C
TFEB	transcription factor EB
GSEA	gene set enrichment analysis
KPNA6	Karyopherin subunit alpha 6
MS	Mass spectrometry
NUP	nucleoporin
P53	tumor protein p53
Co-IP	co-immunoprecipitation
ROS	reactive oxygen species
MS	mass spectrometry
CLEAR	coordinated lysosomal expression and regulation
ECM	extracellular matrix
PH1	Pleckstrin homology 1
SOX2	SRY-box transcription factor 2
PARD3	par-3 family cell polarity regulator
FGFR1	fibroblast growth factor receptor 1
JNK	c-Jun N-terminal kinase
ITGAL	integrin subunit alpha L
ITGAX	integrin subunit alpha X
H3K4me3	histone H3 lysine 4 trimethylation
ROS1	ROS proto-oncogene 1
RASGRF1	RAS guanyl releasing factor 1
MEK	MAPK/ERK kinase
RAF	RAF proto-oncogene serine/threonine-protein kinase
SCLC	small-cell lung cancer

## Appendix A

**Table A1:** Moderately characterized TMEMs and their aliases.

Name	Gene Description	Aliases	Log-Rank *p* Value ^1^ (Raw Values)	
			DFS	OS
AGMO	Alkylglycerol monooxygenase	TMEM195	/	/
ANO1	Anoctamin 1	TMEM16A	/	(−)Q:0.016
ANO10	Anoctamin 10	TMEM16K	(−)M:0.00039	(−)M:0.042
ANO2	Anoctamin 2	TMEM16B	/	/
ANO3	Anoctamin 3	TMEM16C	/	/
ANO4	Anoctamin 4	TMEM16D	(−)M:0.046	/
ANO5	Anoctamin 5	TMEM16E	/	/
ANO6	Anoctamin 6	TMEM16F	(−)Q:0.03	(−)Q:0.0058
ANO7	Anoctamin 7	TMEM16G	(−)M:0.012	/
ANO8	Anoctamin 8	TMEM16H	(−)Q:0.011	/
ANO9	Anoctamin 9	TMEM16J	(−)Q:0.042	/
ARHGAP42	Rho GTPase activating protein 42	AD031, GRAF3, TMEM133	/	/
B3GNT3	UDP-GlcNAc:betaGal beta-1,3-N-acetylglucosaminyltransferase 3	B3GAL-T8, B3GN-T3, B3GNT-3, HP10328, TMEM3, beta3Gn-T3	(−)M:3.2 × 10^−5^	(−)Q:0.011
C2CD2	C2 calcium-dependent domain containing 2	C21orf25, C21orf258, TMEM24L	/	/
C2CD2L	C2CD2 like	DLNB23, TMEM24	/	/
CATSPERD	Cation channel sperm-associated auxiliary subunit delta	TMEM146	/	/
CCDC200	Coiled-coil domain containing 200	LINC00854, TMEM106A-AS	/	/
CEMIP	Cell migration inducing hyaluronidase 1	CCSP11, HYBID, KIAA1199, TMEM2L	/	/
CLRN3	Clarin 3	TMEM12, USH3AL1	(−)Q:0.019	(−)Q:0.038
CLTRN	Collectrin, amino acid transport regulator	NX-17, NX17, TMEM27	(−)M: 0.045	/
CNPY2	Canopy FGF signaling regulator 2	TMEM4	(−)M:5 × 10^−4^	/
DOLK	Dolichol kinase	TMEM15	/	/
DRAM2	DNA damage regulated autophagy modulator 2	TMEM77	(+)Q:0.047	(+)Q:0.037
ELP6	Elongator acetyltransferase complex subunit 6	C3orf75, TMEM103	/	/
EMC3	ER membrane protein complex subunit 3	POB; TMEM111	/	/
EMC4	ER membrane protein complex subunit 4	PIG17, TMEM85	/	/
EMC6	ER membrane protein complex subunit 6	RAB5IFL, TMEM93	/	(−)M:0.0023
EVA1A	Eva-1 homolog A, regulator of programmed cell death	FAM176A, TMEM166	(−)Q:0.0027	/
FAM156A	Family with sequence similarity 156 member A	TMEM29	/	/
FAM156B	Family with sequence similarity 156 member B	TMEM29B	/	(+)M:0.035
FAM174A	Family with sequence similarity 174 member A	TMEM157	(−)M:0.0063	/
FAM187B	Family with sequence similarity 187 member B	TMEM162	Error	Error
HGSNAT	Heparan-alpha-glucosaminide N-acetyltransferase	RP73, HGNAT, MPS3C, TMEM76	(−)M:0.0068	(−)M:0.0084
IGFLR1	IGF-like family receptor 1	TMEM149	/	/
LDAF1	Lipid droplet assembly factor 1	TMEM159	/	/
LMF1	Lipase maturation factor 1	TMEM112, TMEM112A	/	/
LMF2	Lipase maturation factor 2	TMEM112B, TMEM153	/	/
MMGT1	Membrane magnesium transporter 1	TMEM32	/	/
NALF2	NALCN channel auxiliary factor 2	CXorf63, FAM155B, TED, TMEM28, bB57D9.1	(+)Q:0.046	/
NDC1	NDC1 transmembrane nucleoporin	NEDAPA, NET3, TMEM48	/	(−)Q:0.017
NEMP1	Nuclear envelope integral membrane protein 1	TMEM194, TMEM194A	/	/
NEMP2	Nuclear envelope integral membrane protein 2	TMEM194B	/	/
OOSP2	Oocyte secreted protein 2	OOSP2A, PLAC1L, TMEM122	Error	Error
OPALIN	Oligodendrocytic myelin paranodal and inner loop protein	TMEM10	Error	Error
OPN5	Opsin 5	TMEM13	Error	Error
ORAI1	ORAI calcium release-activated calcium modulator 1	TMEM142A	/	/
ORAI2	ORAI calcium release-activated calcium modulator 2	TMEM142B	/	/
ORAI3	ORAI calcium release-activated calcium modulator 3	TMEM142C	/	/
PACC1	Proton-activated chloride channel 1	ASOR, C1orf75, PAC, PAORAC, TMEM206, hPAC	/	/
PEDS1	Plasmanylethanolamine desaturase 1	CarF, KUA, TMEM189	(+)M:0.00019	/
PEDS1-UBE2V1	PEDS1-UBE2V1 readthrough	CROC-1B, CROC1B, KUA-UEV, TMEM189-UBE2V1	(+)M:0.037	(−)Q:0.023
PIP4P2	Phosphatidylinositol-4,5-bisphosphate 4-phosphatase 2	TMEM55A	/	/
RBM14	RNA binding motif protein 14	TMEM137	/	/
RNFT2	Ring finger protein, transmembrane 2	TMEM118	(−)Q:0.00037	/
RTP3	Receptor transporter protein 3	LTM1, TMEM7, Z3CXXC3	error	error
RXYLT1	Ribitol xylosyltransferase 1	HP10481, MDDGA10, TMEM5	/	(−)M:0.021
SARAF	Store-operated calcium entry-associated regulatory factor	FOAP-7, HSPC035, TMEM66, XTP3	/	/
SGMS1	Sphingomyelin synthase 1	MOB, MOB1, SMS1, TMEM23, hmob33	(−)Q:0.04	/
SHISA2	Shisa family member 2	TMEM46, C13orf13, PRO28631, WGAR9166, bA398O19.2	/	(+)Q:0.031
SHISA4	Shisa family member 4	C1orf40, TMEM58	(−)Q:0.0043	/
SLC35G1	Solute carrier family 35 member G1	C10orf60, POST, TMEM20	(+)Q:0.0096	/
SLC35G2	Solute carrier family 35 member G2	TMEM22	/	(−)M:0.037
SLC35G3	Solute carrier family 35 member G3	AMAC1, TMEM21A	error	error
SLC35G6	Solute carrier family 35 member G6	AMAC1L3, TMEM21B	(−)M:0.06	/
SLITRK2	SLIT- and NTRK-like family member 2	CXorf1, CXorf2, SLITL1, TMEM257, XLID111, KIAA1854	(−)Q:0.046	/
STIMATE	STIM activating enhancer	TMEM110	/	/
STIMATE-MUSTN1	STIMATE-MUSTN1 readthrough	TMEM110-MUSTN1	/	/
SYNDIG1	Synapse differentiation inducing 1	C20orf39, DSPC2, IFITMD5, TMEM90B	/	/
SYNDIG1L	Synapse differentiation inducing 1 like	CAPUCIN, DSPC1, IFITMD4, SynDIG2, TMEM90A	/	/
TEDDM1	Transmembrane epididymal protein 1	EDDM9, Epdd1, HE9, HEL-S-45e, TMEM45C	(+)M:0.05	/
TEX2	Testis expressed 2	HT008, TMEM96	/	/
TLCD4	TLC domain containing 4	TMEM56, FLJ31842	(−)M: 0.03	/
TLCD4-RWDD3	TLCD4-RWDD3 readthrough	TMEM56-RWDD3	(−)M:0.0087	/
TLCD5	TLC domain containing 5	TMEM136	/	/
TPRA1	Transmembrane protein adipocyte associated 1	GPR175, TPRA40, TMEM227	(+)M:0.035	/
TRAPPC10	Trafficking protein particle complex subunit 10	TMEM1	/	/
VMP1	Vacuole membrane protein 1	EPG3, TANGO5, TMEM49	/	/
WDR82	WD repeat domain 82	TMEM113	/	/

^1^ “/” indicates *p* ≥ 0.05 (not statistically significant); M indicates a *p* value calculated using the median cut-off point; Q indicates a *p* value calculated using the quartile cut-off point. For each gene, the cut-off that produced the smaller log-rank *p* value is presented. Direction in parentheses: “+” indicates positive correlation between high expression and longer survival, and “−” indicates negative correlation.

**Table A2:** Unfiltered catalogue of TMEM genes showing any significant survival association (*p* < 0.05) in LUAD and LUSC GEPIA2 analyses, including directionally discordant cases.

Log-Rank *p* Value ^1^		Raw Values		Normalized by Reference Genes					
				GAPDH		ACTB		GNAS	
Name	Aliases	DFS	OS	DFS	OS	DFS	OS	DFS	OS
TMEM107	GRVS638, JBTS29, MKS13, PRO1268	(−)M:8 × 10^−6^	/	(−)Q:0.042	(+)Q:0.014	(−)M:0.039	/	(−)Q:0.0018	/
TMEM11	C17orf35, PM1, PMI	/	(−)Q:0.014	/	(+)M:0.029	(+)Q:0.033	/	/	/
TMEM115	PL6	(−)Q:0.027	/	/	(+)M:0.032	(+)M:0.0047	(+)M:0.0035	/	/
TMEM125	—	(−)M:0.018	/	(−)M:0.028	/	(−)M:0.042	(+)Q:0.024	(−)M:0.0013	/
TMEM127	—	(−)Q:0.02	(−)Q:0.04	/	/	/	/	/	/
TMEM129	D4S2561E	(−)M:0.03	/	/	(+)M:0.049	/	/	/	/
TMEM135	PMP52	(−)M:0.029	/	/	/	/	/	/	/
TMEM140	—	(−)Q:0.0057	(−)Q:0.0077	/	/	/	/	/	/
TMEM14C	C6orf53, HSPC194, MSTP073, NET26, bA421M1.6	(−)M:0.011	/	/	(+)M:0.0039	/	/	(−)Q:0.023	/
TMEM150A	TM6P1, TMEM150, TTN1	(−)Q:2 × 10^−4^	/	(−)M:0.015	(+)M:0.049	/	(+)M:0.0013	(−)Q:0.0025	/
TMEM161B	FLB3342, PRO1313	(−)M:0.0097	/	/	(+)Q:0.026	/	/	/	/
TMEM163	DC29, HLD25, SLC30A11, SV31	/	(+)Q:0.017	/	(+)Q:0.0043	/	(+)Q:0.0036	(−)M:0.05	(+)Q:0.013
TMEM170A	TMEM170	(−)M:0.012	(−)Q:0.013	/	/	/	/	/	/
TMEM171	PRP2	/	(−)M:0.017	/	/	(+)Q:0.018	/	/	/
TMEM175	—	/	/	/	(+)Q:0.021	/	(+)M:0.003	(−)M:0.031	/
TMEM184C	TMEM34, SLC51C3	/	(−)Q:0.014	/	/	(+)M:0.035	/	/	/
TMEM187	CXorf12, DXS9878E, ITBA1	/	/	(−)M:0.023	(+)Q:0.016	/	(+)Q:0.035	/	/
TMEM19	—	(+)M:0.029	(−)Q:0.039	/	/	(+)Q:0.0041	/	/	/
TMEM192	—	/	(−)M:0.0047	/	/	/	/	/	/
TMEM200B	TTMB	/	(−)Q:0.025	/	(+)M:0.026	/	/	/	/
TMEM205	UNQ501	(−)M:0.01	/	/	(+)Q:0.033	/	(+)M:0.02	/	/
TMEM219	IGFBP-3R, IGFBP3R	(−)M:0.0037	/	/	(+)M:0.038	/	/	(−)M:0.029	/
TMEM222	C1orf160, NEDMOSBA	(−)Q:0.01	/	/	(+)Q:0.038	/	/	/	/
TMEM223	Mrx15	(−)M:0.031	/	/	(+)M:0.0045	/	/	/	/
TMEM228	ENTREP2, FAM189A1	(−)Q:0.031	/	/	(+)Q:0.01	(+)M:0.029	(+)Q:0.0068	/	/
TMEM230	C20orf30, HSPC274, dJ1116H23.2.1	/	(−)Q:0.021	/	/	/	/	/	/
TMEM231	ALYE870, JBTS20, MKS11, PRO1886	/	(−)M:0.04	/	/	(+)Q:0.047	/	/	/
TMEM233	DSPB2, IFITMD2	(−)Q:0.033	/	/	(+)Q:0.014	/	(+)Q:0.001	/	/
TMEM240	C1orf70, SCA21	(−)Q:0.046	(−)Q:0.04	/	/	/	/	/	/
TMEM256	C17orf61	(−)Q:0.029	/	/	(+)M:0.018	/	/	/	/
TMEM258	C11orf10, Kud, Kuduk	(−)Q:0.024	Q:0.0058	/	(+)M:0.031	/	/	/	/
TMEM30A	C6orf67, CDC50A	(−)Q: 0.015	/	/	(+)Q:0.047	/	/	/	/
TMEM33	1600019D15Rik, Pom33, SHINC-3, SHINC3	/	(−)Q:0.011	/	/	/	/	/	/
TMEM38B	C9orf87, D4Ertd89e, OI14, TRIC-B, TRICB, bA219P18.1	/	(−)M:0.041	/	/	/	/	/	/
TMEM39B	—	/	/	/	(+)M:0.013	(+)M:8.9 × 10^−5^	/	/	/
TMEM41B	—	(−)M:0.0064	/	/	/	/	/	/	/
TMEM50A	IFNRC, SMP1	(−)Q:0.0011	/	/	(+)M:0.046	/	(+)Q:0.034	(−)Q:0.04	/
TMEM52B	C12orf59	(−)Q:0.049	/	/	(+)Q:0.026	(+)M:0.032	(+)Q:0.018	/	/
TMEM53	CTDI, NET4	(−)Q:0.017	/	(−)M:0.024	(+)Q:0.028	/	(+)M:0.011	(−)Q:0.0094	/
TMEM59	C1orf8, DCF1, HSPC001, PRO195, UNQ169	(−)Q:0.023	/	/	(+)Q:0.033	/	(+)Q:0.016	(−)Q:0.031	/
TMEM5	RXYLT1, HP10481, MDDGA10	/	(−)M: 0.021	/	/	(+)M:0.035	/	/	/
TMEM62	—	(−)M:0.0065	/	(−)M:0.0075	(+)Q:0.033	/	(+)Q:0.0087	(−)M:0.0021	/
TMEM63A	HLD19, KIAA0792	(−)M:0.00023	/	(−)Q:0.0076	(+)M:0.0076	(−)Q:0.013	(+)Q:0.039	(−)M:0.0012	/
TMEM65	—	/	(−)Q:0.032	/	(+)Q:0.03	/	/	/	/
TMEM74	NET36	(−)Q:0.041	/	/	(+)Q:0.0053	/	(+)M:0.02	/	/
TMEM80	—	(−)M:0.011	/	/	(+)Q:0.039	/	(+)Q:0.022	(−)Q:0.018	/
TMEM86A	—	(−)Q:0.017	/	/	(+)M:0.013	/	(+)M:0.036	/	/
TMEM9	TMEM9A, DERM4, DERM4A	(−)M:0.006	/	/	/	/	/	/	/
TMEM9B	C11orf15	(−)Q:0.0087	/	/	/	/	(+)Q:0.025	/	/
TMEM98	TADA1	(−)M:0.037	/	/	(+)M:0.021	/	(+)M:0.049	(−)Q:0.036	(+)M:0.02

^1^ “/” indicates *p* ≥ 0.05 (not statistically significant); M indicates a *p* value calculated using the median cut-off point; and Q indicates a *p* value calculated using the quartile cut-off point. For each gene, the cut-off that produced the smaller log-rank *p* value is presented. Direction in parentheses: “+” indicates positive correlation between high expression and longer survival, and “−” indicates negative correlation.
